# Hyperoxia exposure upregulates Dvl-1 and activates Wnt/β-catenin signaling pathway in newborn rat lung

**DOI:** 10.1186/s12860-023-00465-6

**Published:** 2023-02-02

**Authors:** Yuting Zhu, Yawen Li, Weilai Jin, Zhengying Li, Le Zhang, Yuanyuan Fang, Yanyu Zhang

**Affiliations:** Department of Neonatology, The Affiliated Wuxi Children’s Hospital of Nanjing Medical University, No. 299-1 Qingyang Road, Wuxi, 214023 China

**Keywords:** Hyperoxia, Lung injury, Dvl-1, Wnt/β-catenin, Bronchopulmonary dysplasia, Alveolar typeII epithelial cell

## Abstract

**Background:**

Bronchopulmonary dysplasia is a serious and lifelong pulmonary disease in premature neonates that influences around one-quarter of premature newborns. The wingless-related integration site /β-catenin signaling pathway, which is abnormally activated in the lungs with pulmonary fibrosis, affects cell differentiation and lung development.

**Methods:**

Newborn rats were subjected to hyperoxia exposure. Histopathological changes to the lungs were evaluated through immunohistochemistry, and the activation of disheveled and Wnt /β-catenin signaling pathway components was assessed by Western blotting and real-time PCR. The abilities of proliferation, apoptosis and migration were detected by Cell Counting Kit-8, flow cytometry and scratch wound assay, respectively.

**Results:**

Contrasting with normoxic lungs, hyperoxia-exposed lungs demonstrated larger alveoli, fewer alveoli and thicker alveolar septa. Superoxide dismutase activity was significantly decreased (7th day: *P* < 0.05; 14th day: *P* < 0.01) and malondialdehyde significantly increased (7th day: *P* < 0.05; 14th day: *P* < 0.01) after hyperoxia exposure. Protein and mRNA expression levels of β-catenin, Dvl-1, CTNNBL1 and cyclin D1 were significantly upregulated by hyperoxia exposure on 7th day (*P* < 0.01) and 14th day (*P* < 0.01). In hyperoxic conditions, Dvl-l downregulation and Dvl-l downregulation + MSAB treatment significantly increased the proliferation rates, decreased the apoptosis rates and improved the ability of cell migration. In hyperoxic conditions, Dvl-l downregulation could decrease the mRNA expression levels of GSK3β, β-catenin, CTNNBL1 and cyclin D1 and decrease the protein relative expression levels of GSK3β, p-GSK3β, β-catenin, CTNNBL1 and cyclin D1.

**Conclusions:**

We confirmed the positive role of Dvl-1 and the Wnt/β-catenin signaling pathway in promoting BPD in hyperoxia conditions and provided a promising therapeutic target.

**Supplementary Information:**

The online version contains supplementary material available at 10.1186/s12860-023-00465-6.

## Introduction

Bronchopulmonary dysplasia (BPD) is a serious and lifelong pulmonary disease in premature neonates that impacts a quarter of premature newborns [1–3] . Preterm birth is related to an increased risk of long-term pulmonary problems. The pathogenesis of BPD involves a complex interaction between genetic and environmental factors. A variety of endogenous and exogenous stimuli, such as ventilator volume injury, barotrauma, hyperoxia injury and patent ductus arteriosus, can result in inflammatory cascade reaction, immature lung tissue, uncontrolled pulmonary vascular development and abnormal repair of lung tissue [4, 5]. Oxygen therapy is extensively applied for the treatment of BPD. However, hyperoxia causes many adverse effects, and the lung is generally the first damaged organ [6–8]. Preterm birth is also associated with a high risk of respiratory distress syndrome, which is caused by immaturity of alveolar type II epithelial cells (AECII) [9].

Current research shows that the Wnt/β-catenin signaling pathway can regulate the normal development of multiple organs in the body. It is important that the discovery of its molecular mechanism in the lung, using targeted therapy to treat BPD without causing damage to other organs. Dishevelled-1 (Dvl-1) had been reported to mediate three signaling pathways, including the canonical Wnt/β-catenin signaling pathway, the non-canonical Wnt/β-catenin signaling pathway and Wnt/Ca^2+^ signaling pathway [10]. In the canonical Wnt/β-catenin signaling pathway, the upregulation of Dvl-1 protein expression leads to the overexpression of β-catenin protein. The accumulation of β-catenin protein in the cytoplasm promotes the transcription of the Wnt/β-catenin signaling pathway downstream genes in the nucleus [11]. Recent studies have showen that abnormal activation of the Wnt/β-catenin signaling pathway causes the occurrence of pulmonary diseases [12, 13]. In the newborn’s lung, the Wnt/β-catenin signaling pathway is activated in pulmonary fibrosis, affecting abnormal cell differentiation and tissue structure [14]. The Wnt/β-catenin signaling pathway was activated in the lungs of BPD animal models and idiopathic pulmonary fibrosis, which suggested that Wnt/β-catenin signaling pathway has potential as a therapeutic target of BPD [15, 16].

In this study, we explore the role of Dvl-1 and the Wnt/β-catenin signaling pathway in the occurrence and development of BPD. It is demonstrated that the downregulation of Dvl-l relieves hyperoxia-induced AECII apoptosis by inhibiting the Wnt/β-catenin signalling pathway. Our study provides a new perspective and theoretical basis for the clinical treatment of hyperoxia-induced lung injury through Dvl-1. It is helpful to supple treatment of hyperoxia lung injury except supportive therapy currently.

## Results

### Hyperoxia-induced pathological changes in the lung

**Fig. 1:**
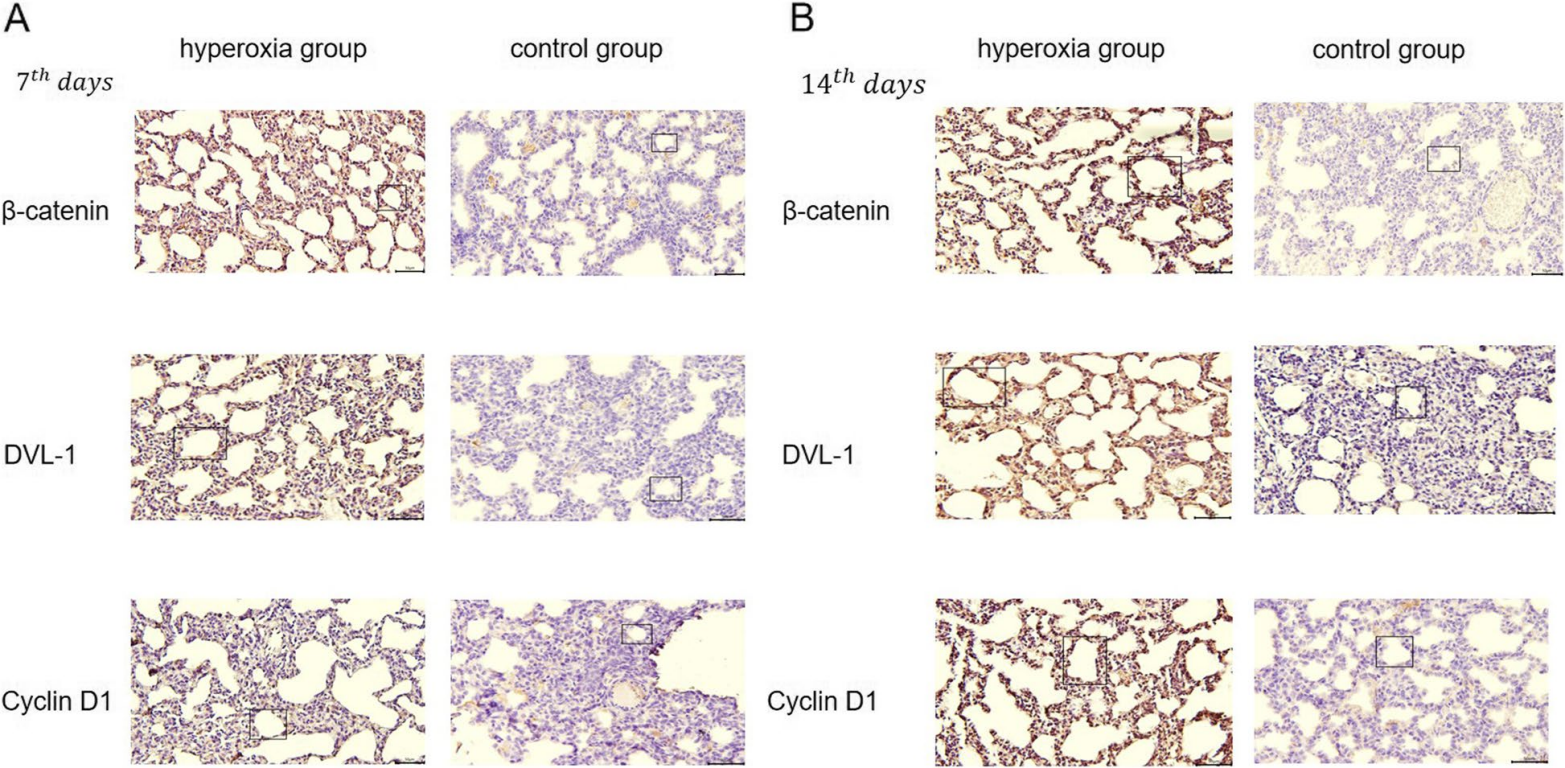
Hyperoxia exposure could increase the expression of β-catenin, DVL-1 and CYCLIN D1 in the lung. **A** Hyperoxic exposure at 7th day (magnification × 20), **B** hyperoxic exposure at 14th day (magnification × 20)

In the present study, rats exposed to 85% O2 had inhibited alveolar development, which was correlated with the pulmonary morphological changes of BPD (Fig. 1). A minimum of nine fields of view for each slice was examined. On the 7th day, the alveoli of the hyperoxia-induced rats were separated irregularly. Contrasting with the normoxic lungs, the hyperoxia-exposed lungs demonstrated larger alveoli, fewer alveoli and thicker alveolar septa. On the 14th day, the number of alveoli reduced obviously and enlarged significantly in the hyperoxia group.

In the hyperoxia group, IHC scores of β-catenin, Dvl-1, and cyclin D1 were significantly higher than those of the control group on the 7th day (*P* < 0.01) and were also significantly higher on the 14th day than those on the 7th day(*P* < 0.01) (Fig. 1, Table 1). SOD, the main antioxidant enzyme, and MDA, an end-product of membrane lipid peroxidation, were used as indicators of cell oxidation. We found that SOD activity was significantly decreased (7th day: *P* < 0.05; 14th day: *P* < 0.01) and MDA was significantly increased (7th day: *P* < 0.05; 14th day: *P* < 0.01) after hyperoxia exposure as shown in Table 2.

**Table 1:** Results of immunohistochemical staining

Target protein	7th day IHC scores		14th day IHC scores	
	Hyperoxia group	Control group	Hyperoxia group	Control group
β-catenin	3.42 ± 0.38^**^	1.56 ± 0.29	5.26 ± 0.92^**^	1.54 ± 0.37
Dvl-1	3.51 ± 0.42^**^	1.49 ± 0.35	5.84 ± 0.89^**^	1.48 ± 0.76
cyclin D1	3.70 ± 0.91^**^	1.51 ± 0.46	5.78 ± 0.84^**^	1.69 ± 0.86

^**^P < 0.01 versus control group

**Table 2:** Oxidative stress on hyperoxia exposure lungs (mean ± SD, *n* = 9)

Parameter	Hyperoxia group		Control group	
	7th day	14th day	7th day	14th day
MDA (mmol/g prot)	0.71 ± 0.16^*^	1.38 ± 0.31^**^	0.61 ± 0.11	0.65 ± 0.24
SOD (U/g prot)	24.76 ± 3.85^*^	18.86 ± 4.36^**^	55.47 ± 4.39	52.92 ± 3.96

^*^P < 0.05 versus control group; *P* < 0.01 versus control group^**^

### Hyperoxia-exposure upregulated Dvl-1 protein expression and activated Wnt/β-catenin signaling pathway in newborn rat lung

**Fig. 2:**
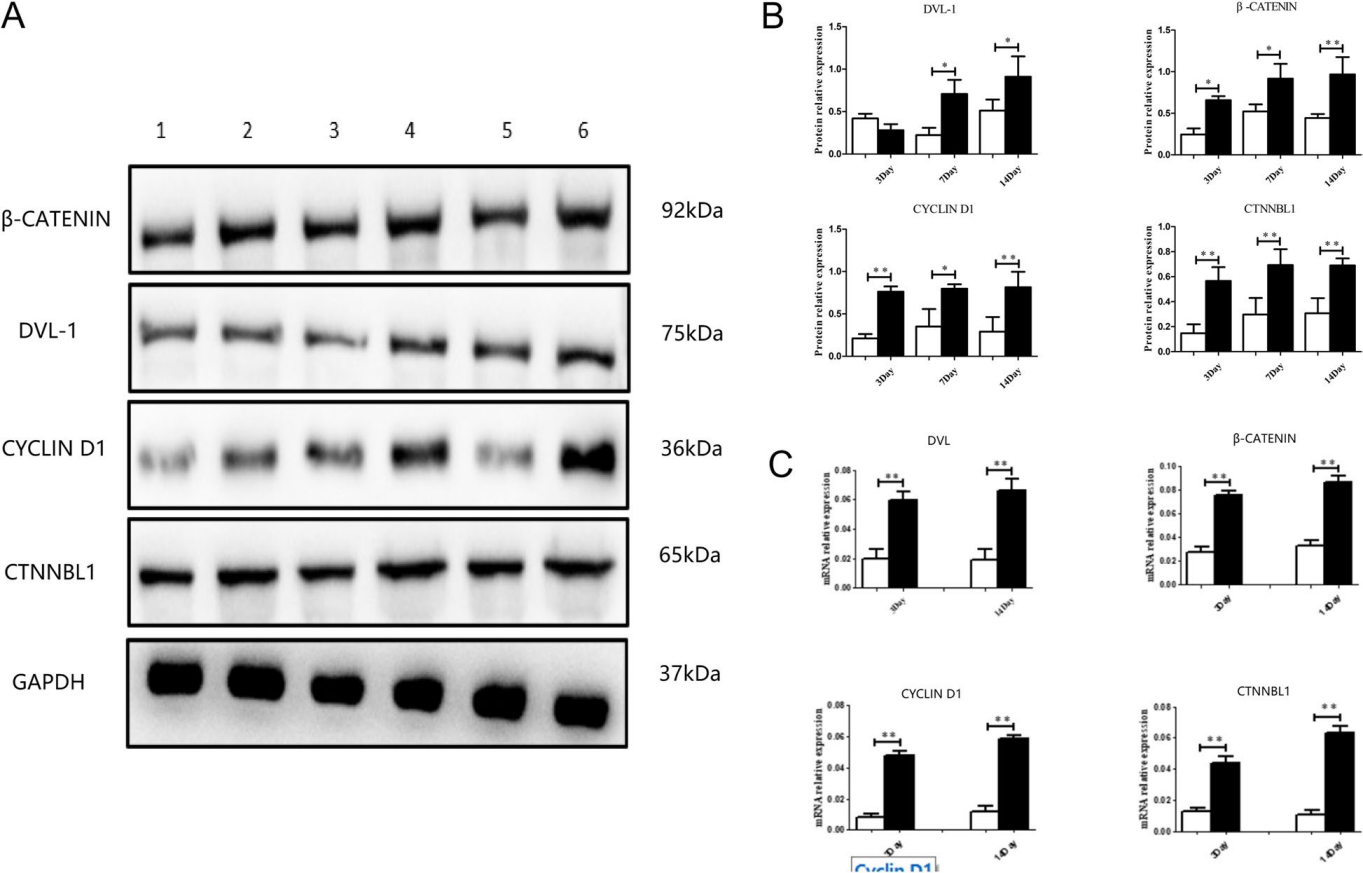
Hyperoxia-exposure upregulated Dvl-1 protein expression and activated Wnt/β-catenin signaling pathway in newborn rat lung. **A** Expression of β-catenin, Dvl-1, CTNNBL1 and cyclin D1 proteins levels by western blot 1: normoxia exposure on 3rd day, 2: hyperoxia exposure on 3rd day, 3: normoxia exposure on 7th day, 4: hyperoxia exposure on 7th day, 5: normoxia exposure on 14th day, 6: hyperoxia exposure on 14th day. **B** Normalized against GAPDH. **C** Expression of β-catenin, Dvl-1, CTNNBL1 and cyclin D1 mRNA levels by real-time PCR and normalized against Gapdh, *p < 0. 05, **p < 0. 01 was considered statistically significant

As shown in Fig. 2, compared with the control group, the protein expression levels of Dvl-1 were significantly upregulated after hyperoxia exposure on the 7th day and the 14th day (*P* < 0.01), but there was no significant difference after hyperoxia exposure on the 3rd day. The protein expression levels of β-catenin, CTNNBL1 and cyclin D1 were significantly upregulated after hyperoxia exposure on the 3rd day(*P* < 0.01), the 7th day(*P* < 0.01) and the 14th day(*P* < 0.01) .

The results of the real-time PCR analysis indicated that the mRNA expression levels of β-catenin, Dvl-1, CTNNBL1 and cyclin D1 were significantly upregulated by hyperoxia exposure on the 7th day (*P* < 0. 01) and on the 14th day (*P* < 0. 01).

### Dvl-l downregulation attenuated the inhibition of hyperoxia-induced cell proliferation and migration in ACEII cells

**Fig. 3:**
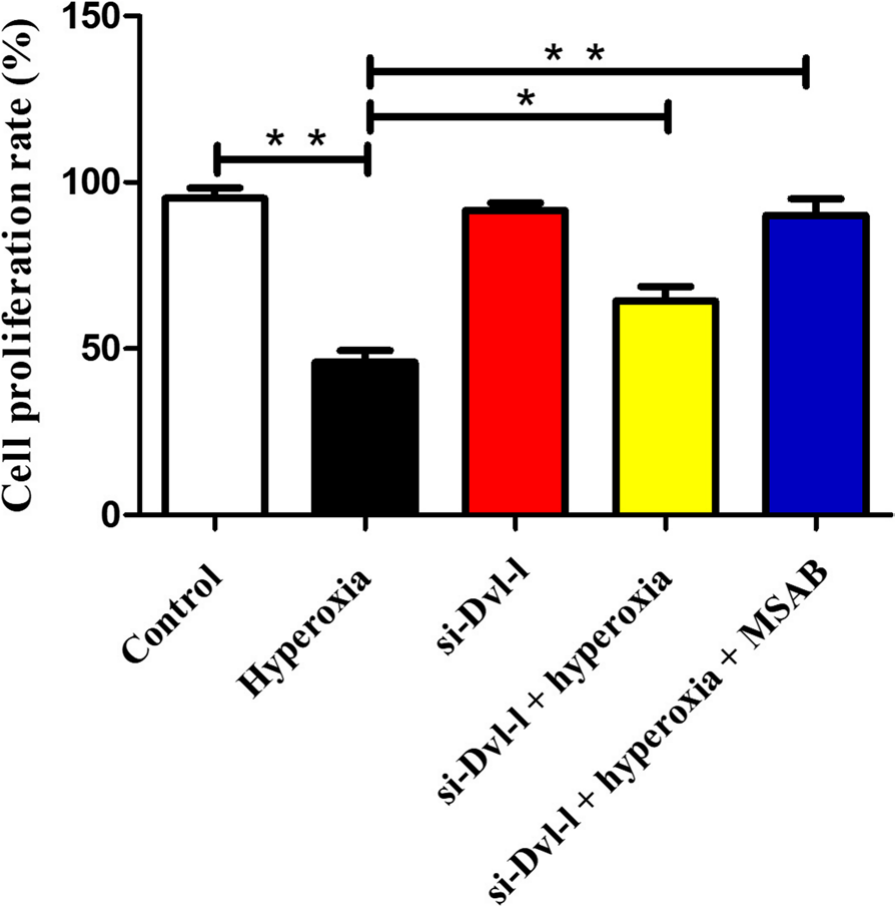
Dvl-1 downregulation attenuates the inhibition of hyperoxia-induced cell proliferation in ACEII cells. * *p* < 0.05 and ** *p* < 0.01 was considered statistically significant

We examined the cell proliferation and motility by CCK-8 assay and scratch wound assay, respectively. Under hyperoxic conditions, the proliferation of ACEII cells was inhibited. However, Dvl-l downregulation treatment increased the cell proliferation rate of ACEII cells. Dvl-l downregulation could reverse the hyperoxia-induced inhibition effect on ACEII cells proliferation. It is noteworthy that the promoting proliferation effect of Dvl-l downregulation can be enhanced by MSAB, which is a selective inhibitor of the Wnt/β-catenin signaling pathway [17]. It also indicated that Dvl-l downregulation promoted proliferation via the inhibition of the Wnt/β-catenin signaling pathway, which suggests that Dvl-l plays a negative role in ACEII cells proliferation. The proliferation rates with downregulated Dvl-l and MSAB under hyperoxic conditions were significantly higher than those of the hyperoxia group (*P* < 0.01) but there was no significant difference in Dvl-l downregulation between the hyperoxia condition group and the control group (*P* > 0.05) (Fig. 3).

**Fig. 4:**
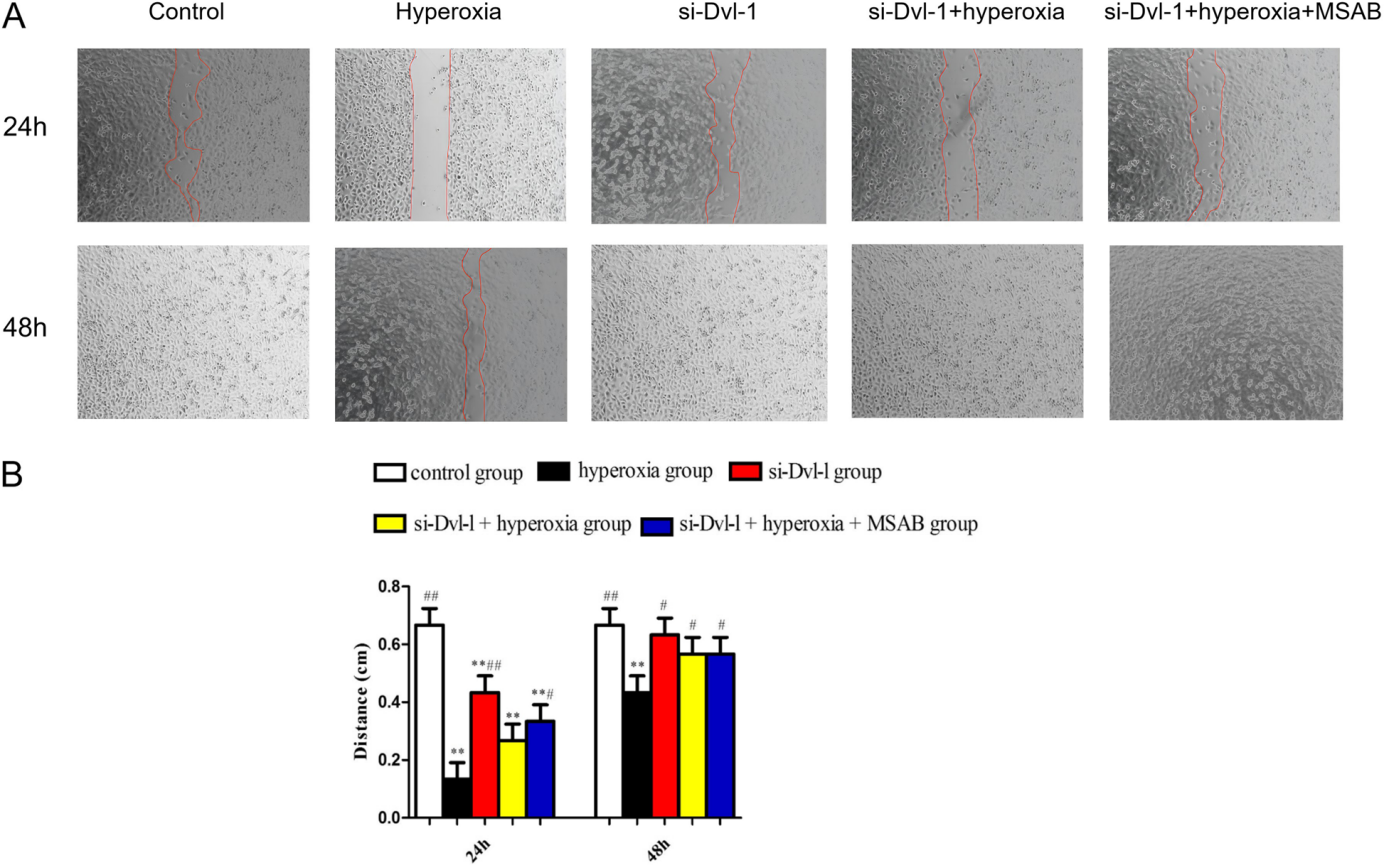
Dvl-1 downregulation attenuates the inhibition of hyperoxia-induced cell migration in ACEII cells. **A** Representative images of wound healing assay for ACEII cells at 24 h and 48 h. Red lines indicate the leading edge of the migrating cells (magnification × 40). **B** Migration of ACEII cells was tested and the distance of the wound was calculated up to 48 h. Compared with control group, *p* < 0.01 was considered statistically significant; compared with hyperoxia group, *p* < 0.05 and *p* < 0.01 was considered statistically significant^**^^#^^##^

In the scratch wound assay, we drew solid red lines to indicate the edges of the scratch wounds. After 24 h and 48 h of hyperoxia exposure, the hyperoxia inhibited ACEII cell migration (*P* < 0.01). After 24 h of hyperoxia exposure, Dvl-l downregulation and MSAB treatment improved ACEII cell migration (*P* < 0.05), which indicated that the combined treatment of Dvl-l downregulation and MSAB relieved hyperoxia-induced inhibition of ACEII cell migration (Fig. 4).

### Dvl-l downregulation ameliorated hyperoxia-induced ACEII cells apoptosis

**Fig. 5:**
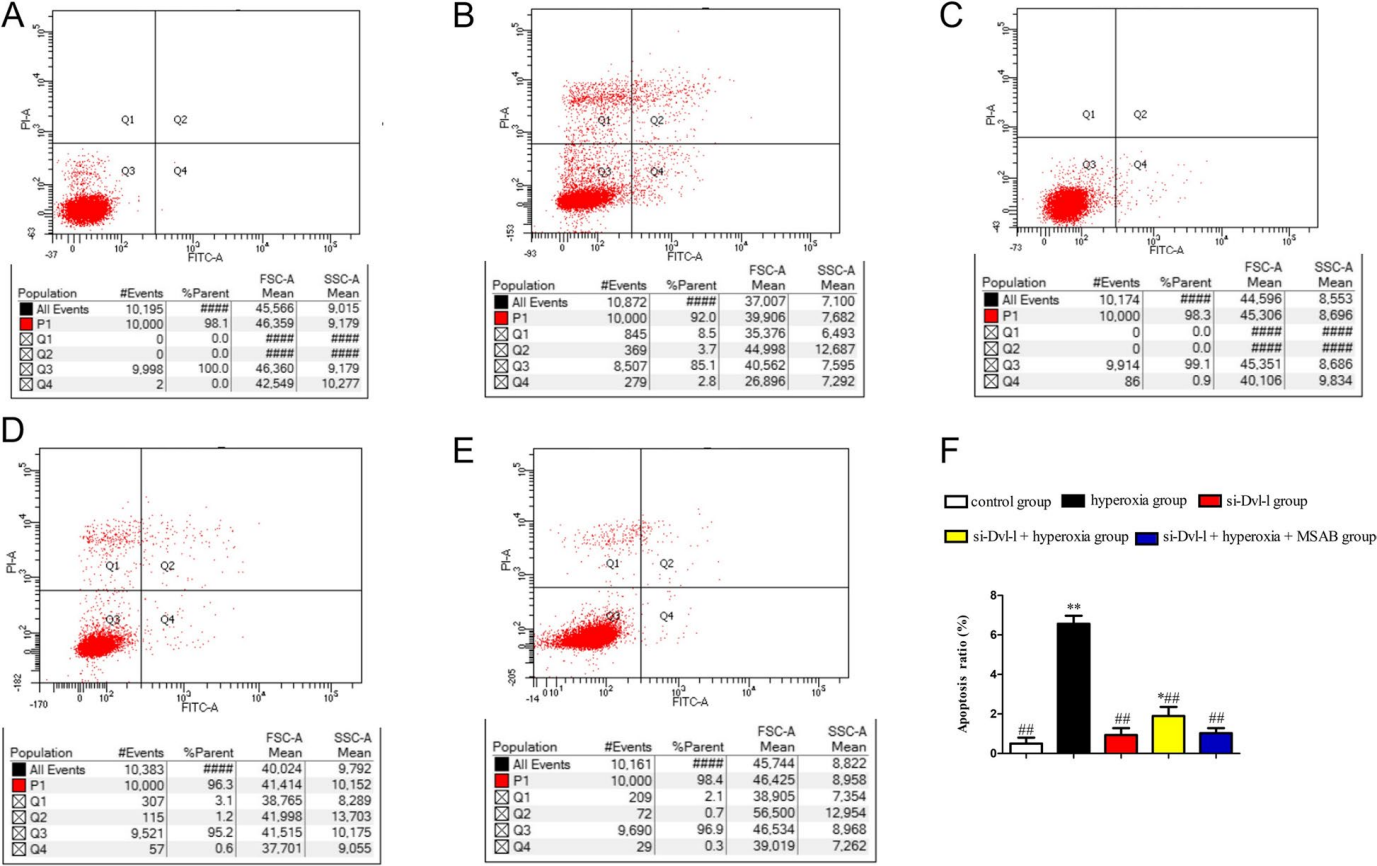
Dvl-1 downregulation ameliorated hyperoxia-induced ACEII cells apoptosis. **A** Control, **B** Hyperoxia, **C** si-Dvl-l, **D** si-Dvl-l + hyperoxia, **E** si-Dvl-l + hyperoxia + MSAB, **F** Quantitative analysis of apoptotic cell percentages. Percentage of cell apoptosis = Q2 (early apoptosis) + Q4 (late apoptosis). Compared with control group, *p* < 0.05 and *p* < 0.01 was considered statistically significant; compared with hyperoxia group, *p* < 0.01 was considered statistically significant^*^^**^^##^

We also detected hyperoxia-induced apoptosis by flow cytometry with annexin V and PI dual staining. The apoptosis rate was equal to the sum of the late apoptosis rate (Q2) and the early apoptosis rate (Q3). After transient transfection and subsequent culture for 24 h, the apoptosis rate of the hyperoxia group was significantly higher than that of the control group (*P* < 0.01) which suggested that hyperoxia could induce ACEII cells apoptosis. However, a reverse effect occurred in the si-Dvl-l, si-Dvl-l + hyperoxia and si-Dvl-l + hyperoxia + MSAB groups, in which the cell apoptosis rates were significantly lower than they were in the hyperoxia group (*P* < 0.01). Although the apoptosis rate of the si-Dvl-l + hyperoxia group was lower than that of the si-Dvl-l + hyperoxia + MSAB group, there was no significant difference(*P* > 0.05). Our results indicated that Dvl-l downregulation inhibited hyperoxia-induced apoptosis (Fig. 5).

### Dvl-l downregulation inhibited Wnt/β-catenin signaling pathway in ACEII cells

**Fig. 6:**
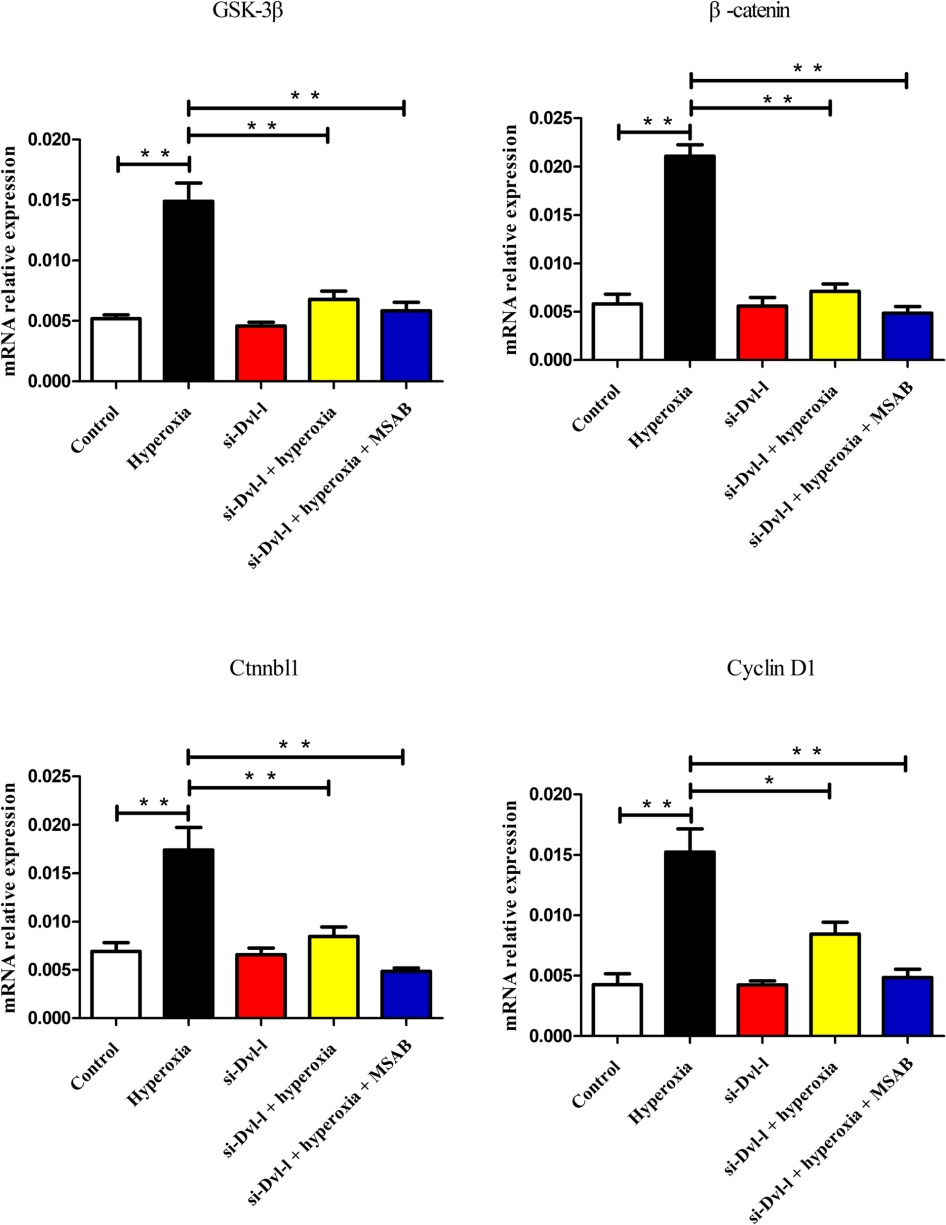
Dvl-1 downregulation inhibited the mRNA expression of Wnt/β-catenin signaling pathway related genes in ACEII cells. * *p* < 0.05 and ** *p* < 0.01 was considered statistically significant

The result of real-time PCR analysis indicated that the mRNA expression levels of GSK-3β, β-catenin, CTNNBL1, and cyclin D1 were upregulated by hyperoxia exposure. Compared with the control group, there was GSK3β, β-catenin, CTNNBL1, and cyclin D1 upregulation in the hyperoxia group at 24 h after hyperoxia exposure (*P* < 0.01). Compared with the hyperoxia group, the mRNA relative expression levels of GSK3β, β-catenin, CTNNBL1, and cyclin D1 in the si-Dvl-l + hyperoxia and si-Dvl-l + hyperoxia + MSAB groups were significantly deceased (Fig. 6).

**Fig. 7:**
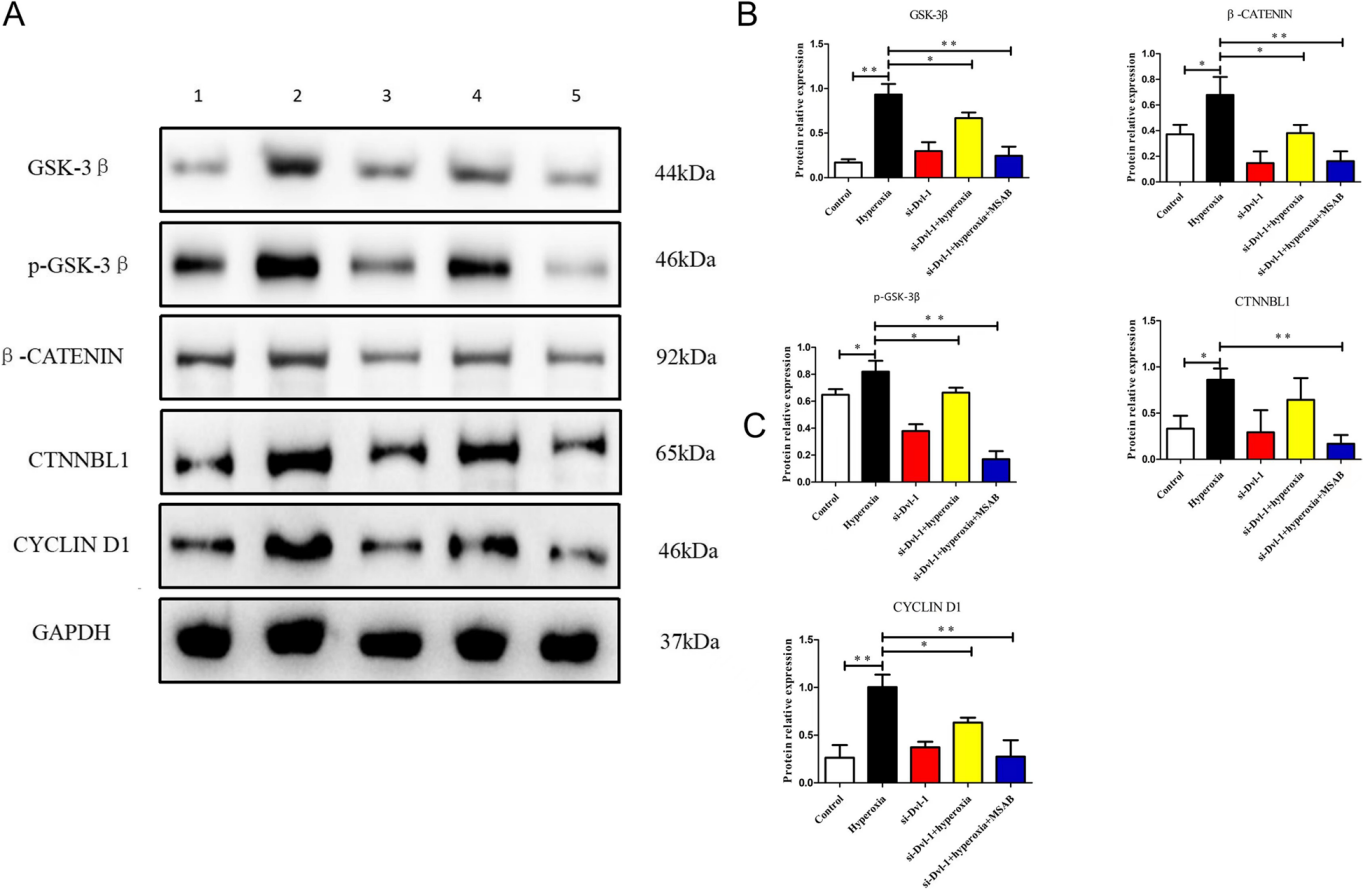
Dvl-1 downregulation inhibited the protein expression of Wnt/β-catenin signaling pathway related genes in ACEII cells. **A** Expression of Wnt/β-catenin signaling pathway proteins levels by western blot, 1: Control, 2: Hyperoxia, 3: si-Dvl-1, 4: si-Dvl-1+ Hyperoxia, 5: si-Dvl-1+ Hyperoxia+ MSAB. **B** normalized against GAPDH. * *p* < 0.05 and ** *p* < 0.01 was considered statistically significant

Consistent with the real-time PCR results, Western blotting analysis demonstrated significant increases in GSK-3β, p-GSK3β, β-catenin, CTNNBL1 and cyclin D1 protein levels in hyperoxia-treated cells at 24 h (*P* < 0.01). We also assessed the effect of Dvl-l downregulation on the above protein expressions in hyperoxia conditions. Dvl-l downregulation could decrease the protein expression levels of GSK3β, p-GSK3β, β-catenin, CTNNBL1 and cyclin D1 (*P* < 0.01). Interestingly, MSAB, a Wnt/β-catenin signaling pathway inhibitor, enhanced the inhibition effect of Dvl-l downregulation on the protein expression levels of GSK3β, p-GSK3β, β-catenin, CTNNBL1 and cyclin D1. These results of real-time PCR and Western blotting analysis collaboratively suggested that Dvl-l downregulation could inhibit hyperoxia-induced Wnt/β-catenin signaling pathway activation in ACEII cells (Fig. 7).

## Discussion

There is mounting evidence from laboratory animal research and clinical observations that neonatal hyperoxia leads to injury in some organs, especially the lung, and leads to BPD [18, 19]. BPD, one of the most common sequelae of premature birth, is a chronic lung disease that is characterized by dysplasia of pulmonary alveoli and pulmonary microvascular. Neonatals with BPD are prone to growth and mental disorders that will increase the risk of asthma and lower respiratory tract infection during the growth process. Using the model of hyperoxia-induced lung injury to simulate BPD to find its mechanism, the therapeutic target of BPD will help guide the clinical prevention and treatment of BPD.

Dvl-1 is the crucial regulator of the Wnt/β-catenin signaling pathway, which is associated with the organogenesis, tissue homeostasis, and pathogenesis of many human diseases. The canonical Wnt/β-catenin signaling pathway is most sensitive to the changes in the abundance of Dvl-3 or Dvl-1 [20]. Previous studies have confirmed that Dvl-1 promots the occurrence and development of lung tumors. Coexpression of Dvl-1 and IQ-domain GTPase-activating protein 1 (IQGAP1) in the cytoplasm and nucleus is closely related to the poor prognosis of non-small-cell lung cancer. Coexpression in the nucleus might play a critical role in the activation of the canonical Wnt/β-catenin signaling Wnt pathway [21]. Phosphorylated dishevelled-2 (Dvl-2) protein is significantly higher in cisplatin resistant A549 cells [22]. However, there has been no report on the mechanism of Dvl-1 involved in hyperoxia-induced lung injury or BPD. In our experiment, we found that the protein expression level of Dvl-1 was significantly up-regulated on the 7th and 14th days under hyperoxia.

Recent studies have shown that the Wnt/β-catenin signaling pathway plays an extremely important role in the growth of the lung and the occurrence and development of lung diseases [23–25]. When the Wnt/β-catenin signaling pathway is abnormally activated, it will lead to abnormal lung development. Transforming growth factor-β upregulates canonical Wnt signaling and inhibits the peroxisome proliferator activated receptor gamma (PPARγ). The absence or inhibition of Wnt/β-catenin signaling, which is partly related to inflammatory processes during the canalicular stage of pulmonary development, severely affects the developmental processes during the subsequent saccular and alveolar stages. PPARγ stimulates transdifferentiation of myofibroblasts into lipofibroblasts, which helps normal alveolarization. Importantly, hyperoxia activates the canonical Wnt/β-catenin signaling pathway, upregulating Tgf-β expression and downregulating PPARγ expression [25]. The administration of PPARγ agonist has been shown to prevent hyperoxia-induced molecular and morphological changes in rat models. In our study, the protein expression levels of the Wnt/β-catenin pathway were increased after 7 days and 14 days of hyperoxia exposure. Aberrant activation of Wnt/β-catenin pathway induced heterotopic differentiation of the alveoli, increased alveolar volume, and reduced alveolar number.

Under hyperoxic conditions, different signaling pathways have different functions on hyperoxia-induced lung injury. The activation of the PI3K/Akt/FoxO3a pathway protects AECII cells from hyperoxia-induced apoptosis and increases the expression levels of anti-apoptosis factors [26]. Negatively regulating the JNK signaling pathway could promote proliferation and inhibit apoptosis, attenuating oxidative stress damage in AECII cells after hyperoxia exposure [27]. The JAK/STAT signaling pathway is implicated in inflammatory and autoimmune diseases that are positively correlated to lung inflammation [28]. The phosphorylated protein expression level of STAT3 activates the JAK/STAT signaling pathway. In this study, the downregulation of Dvl-l reduced GSK3β, p-GSK3β, β-catenin, CTNNBL1 and cyclin D1 protein expression. Targeting the downregulation of the Dvl-1 gene is helpful for alleviating lung injury caused by hyperoxia. By using the inhibitor of the Wnt/β-catenin signaling pathway and the downregulation of the Dvl-1 gene, it was found that the lung injury caused by hyperoxia was significantly alleviated.

The current research has shown that the Wnt/β-catenin signaling pathway can regulate the normal development of multiple organs in the body. The discovery of its molecular mechanism in the lung will enable the use of targeted therapy to treat BPD without causing damage to other organs. Interestingly, we downregulated Dvl-1, which achieved a similar effect with Wnt/β-catenin signaling pathway inhibitors in AECII cell damage caused by hyperoxia. This study found a new molecular mechanism of the Wnt/β-catenin signaling pathway in promoting the development of BPD based on Dvl-1. The Wnt/β-catenin signaling pathway can be affected by downregulating Dvl-1 to alleviate BPD. In the future, we will study further the targeting treatment BPD with Dvl-1 and the Wnt/β-catenin signaling pathway inhibitor.

## Conclusions

**Fig. 8:**
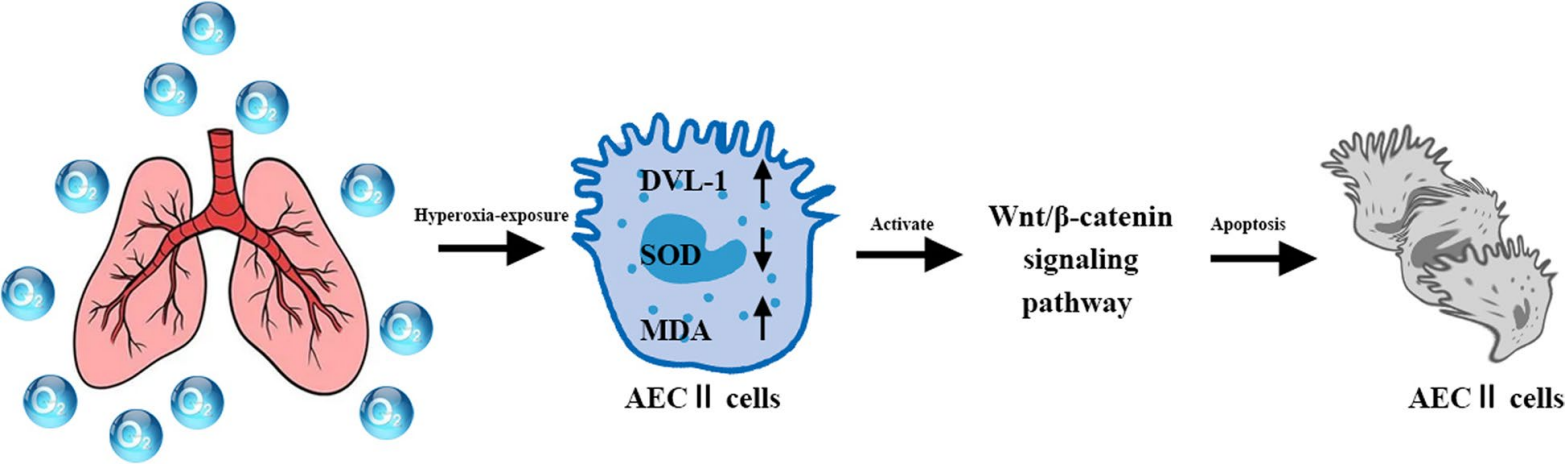
Hyperoxia exposure upregulated the protein expression levels of Dvl-1 and activated Wnt/β-catenin pathway, and induced ACEII apoptosis

Our findings suggest that hyperoxia exposure results in a clearly reduced number of alveoli and significantly enlarged alveoli in the lung tissue of newborn rats. Hyperoxia exposure upregulated the protein expression levels of Dvl-1 and activated the Wnt/β-catenin signaling pathway. Hyperoxia exposure increased GSK3β, p-GSK3β, β-catenin, CTNNBL1 and cyclin D1 protein expression. Dvl-l downregulation had a protective role in hyperoxia-induced ACEII apoptosis. We innovatively revealed that the beneficial effect of Dvl-l downregulation for BPD is achieved by inhibiting the activation of the Wnt/β-catenin pathway (Fig. 8).

## Materials and methods

### Reagents and antibodies

The following were obtained: sheep anti-rabbit horseradish peroxidase (HRP)-conjugated secondary antibody and RIPA lysis buffer (Cell Signaling Technology, MA, USA); BCA protein and, StarSignal chemiluminescent assay kits (Vazyme, Nanjing, China); StarScript II First-strand cDNA Synthesis Mix with gDNA Remover, 2 × RealStar Green Fast Mixture (with ROXII), and TRIGene (TaKaRa, Dalian, China); polyvinylidene difluoride (PVDF) membranes (Millipore, Burlington, USA); GSK3β, p-GSK3β, cyclin D1, Dvl-1, and β-catenin primary antibodies (ZENBIO Biotech, Chengdu, China); GAPDH primary antibody (Goodhere Biotech, Hangzhou, China); PCR primers (Genscript Biotech, Nanjing, China); MDA and SOD assay kits (Solarbio Life Sciences, Beijing, China); immunohistochemistry kit (MXB, Fujian, China), rat AECII cells (Bnbio, Beijing, China); Roswell Park Memorial Institute (RPMI) 1640, trypsin, penicillin, and streptomycin (Gibco, Thermo Fisher Scientific Inc., USA); fetal bovine serum (Biological Industries, Beth Haemek, Israel); lipofectamine 3000 (Invitrogen, Thermo Fisher, USA); AnnexinV-fluorescein isothiocyanate (FITC)/propidium iodide (PI) apoptosis detection kit and Cell Counting Kit-8 (Vazyme, Nanjing, China); and MSAB (Wnt/β-catenin pathway inhibitor (Selleck, Shanghai, China).

### Animal procedures and treatment

All animal experimental procedures were approved by the Ethics Committee of Animalsof the Affiliated Wuxi Children’s Hospital of Nanjing Medical University(No:WXCH2020-04-003 at April 15, 2020). Sprague Dawley (SD) rats were obtained from the Nanjing Agriculture University. A total of 36 newborn SD rats were randomly selected into two groups, hyperoxia group (85% O_2_ from the beginning of birth) and the control group (normoxia, 21% O_2_). The number of rats used in each group is shown in Table 3. Rats had free access to food and water. On the 3rd, the 7th and the 14th days after birth, nine newborn rats from the two groups were anesthetized with sodium pentobarbital, and their lungs were collected. The right lung was fixed with paraformaldehyde (PFA) for immunohistochemistry, the upper lobes of the left lung were used for real-time qPCR and oxidative stress index test, and the lower lobes of the left lung were used for Western blotting.

**Table 3:** Experimental groups

Groups	Number of rats
Control group (the 3rd days after birth)	6
Hyperoxia group (the 3rd days after birth)	6
Control group (the 7th days after birth)	6
Hyperoxia group (the 7th days after birth)	6
Control group (the 14th days after birth)	6
Hyperoxia group (the 14th days after birth)	6

### Oxidative stress index test

The upper lobes of the left lung were homogenized with cold normal saline and centrifuged (4 °C, 12,500 g, 10 min), and the supernatant was collected for assays. MDA and SOD assays were performed using assay kits, according to their manufacturers’ instructions.

### Western blot analysis

Total protein was separated from lung tissues with radio immunoprecipitation assay (RIPA), and quantified with a bicinchoninic acid assay (BCA) protein assay kit. After dilution with loading buffer, the separated proteins were boiled for 5 min. Twenty micrograms of protein samples were separated using 12.5% SDS-PAGE and electrotransferred onto polyvinylidene difluoride membranes. The membranes were probed overnight at 4 °C with primary antibodies against Dvl-1 (1:1000 dilution), β-catenin (1:1000 dilution), GSK3β (1:1000 dilution), p-GSK3β (1:1000 dilution), and CTNNBL1 (1:1000 dilution) and cyclin D1 (1:1000 dilution) and GAPDH (1:1000 dilution) proteins and subsequently hybridized with horseradish peroxidase-conjugated secondary antibodies for 2 h at room temperature. The protein bands were detected using an ECL advanced system (Millipore) and quantified using Photoshop software.

### Immunohistochemistry (IHC)

The lungs were fixed with 4% formalin for 24 h at room temperature. The fixed lungs were sequentially dehydrated, vitrified, embedded in paraffin, fixed, cut into 5 μM thick sections. The sections were dewaxed with dimethylbenzene, hydrated with gradient alcohol according to the manufacturer’s instructions and treated with 3% H_2_O_2_ to block endogenous peroxidase activity. The treated sections were placed into an EDTA-Tris buffer solution and microwaved for 20 min, blocked with serum, and incubated overnight at 4°Cwith Dvl-1 primary antibody (1:200 dilution), β-catenin primary antibody (1:200 dilution) and cyclin D1 primary antibody (1:200 dilution). After sequential incubation with a biotin-labeled secondary antibody and streptavidin-peroxidase, sections were developed using 3,3′-diaminobenzidine (DAB) and dehydrated. IHC score was determined semi-quantitatively by multiplication of the positive fraction with the grayscale value according to the following system: a) positive fraction was categorized as 0 (no staining), 1+ (≤10%), 2+ (>10% and <50%), and 3+ (≥50%); b) intensity was graded as 0 (no staining), 1 (low staining), 2 (medium staining), and 3 (strong staining).

### Real-time polymerase chain reaction (RT-PCR)

Total RNA was extracted using TRIGene reagent according to the manufacturer’s instructions. The purified mRNA was reverse transcribed into cDNA using PrimeScript™ RT reagent kit with gDNA Eraser and real-time PCR was performed using TB Green® Premix Ex Taq™ II (Tli RNaseH Plus). Data were standardized to the endogenous expression of GAPDH. The sequences of the primers are listed in Table 4. Real-time PCR was performed according to the method provided by QuantStudio 3 (Applied Biosystems, USA) in a 20 μl volume using 1 μl cDNA, l μl forward primer, 1 μl reverse primer and 10 μl 2 × RealStar Green Fast Mixture (with ROX II). The thermal cycling conditions were Stage 1 (1 cycle, 30 s at 95 °C), Stage 2 (45 cycles, 10 s at 95 °C, 30 s at 60 °C), and Stage 3 (1 cycle, 15 s at 95 °C, 1 min at 60 °C).

**Table 4:** Sequences of the real-time PCR primers

Gene	Primer sequences	Product length (bp)
GSK-3β	Forward: 5′-TGGGTCATTTGGTGTGG − 3′ Reverse: 5′-CCGCAATCGGACTATGTT-3’	148 bp
β-catenin	Forward: 5’- TATGAGTGGGAGCAAGGC − 3′ Reverse: 5′- CTGCGTGGATGGGATCT − 3’	150 bp
cyclin D1	Forward: 5’- GCGTACCCTGACACCAAT-3′ Reverse: 5′-CTTCGCACTTCTGCTCCT − 3’	178 bp
CTNNBL1	Forward: 5’- AGGTGGTCGCACTATTGG-3′ Reverse: 5′-GCACATCTCTGGACGGA-3’	125 bp
Gapdh	Forward: 5’- CAAGTTCAACGGCACAGTCAAG − 3′ Reverse: 5′- ACATACTCAGCACCAGCATCAC − 3’	123 bp

### Cell culture, transient transfection, and hyperoxia treatment

Rat AECII cells were cultured in RPMI-1640 medium at 37 °C in a 5% CO_2_ atmosphere. The cell culture medium included 10%(v/v) FBS, 100 U/mL penicillin and 100 μg/mL streptomycin. AECII cells were divided into the following five group: AECII cells group (control group), AECII cells hyperoxia exposure group (hyperoxia group), AECII cells Dvl-l downregulation treatment group (si-Dvl-l group), AECII cells Dvl-l downregulation + hyperoxia exposure group (si-Dvl-l + hyperoxia group), and AECII cells Dvl-l downregulation + hyperoxia exposure + 5 μM MSAB treatment group (si-Dvl-l + hyperoxia + MSAB group). When reaching 70% confluency, the transfected AECII cells were treated with lipo3000 and Dvl-l siRNA oligonucleotides and then cultured in serum-free RPMI-1640 medium for 6 h. The cell culture plate was then placed into a sterile modular incubator chamber (MIC-101, Billups-Rothenberg, Del Mar, CA) and exposed to hyperoxia (85% O_2_ and 5% CO_2_) for 24 h.

### Cell counting Kit-8 assay

The proliferation rates of AECII cells in the five groups were detected using CCK-8 assay. AECII cells (1 × 10^5^ cells/mL), after respectively different treatments, were cultured for 24 h, and then prepared as cell suspensions using culture medium. The cell suspension (100 μL) was inoculated into 96-well plates and cultured for 24 h. The solution (10 μL) was added to each well and incubated for 2 h at 37 °C. The absorbance of each well was measured by microplate reader (Shanghai Flash Spectrum Biotechnology, China) at a wavelength of 450 nm. The data analysis was reported in a previous study [29].

### Scratch wound assay

After five different treatments, the AECII cells were cultured for 24 h and then inoculated into 6-well plates and cultured until 100% confluency. A uniform straight scratch was formed in the cells’ monolayer by a 200 μL pipette tip and then washed gently. After culturing for 24 h or 48 h, the scratch wounds were photographed.

### Cell apoptosis analysis

Apoptosis was determined with FITC/PI double staining using a flow cytometer. After different treatments for 24 h, the cells were trypsinized, centrifuged, and washed with cold 1 × PBS. The cells were then resuspended in 200 μL binding buffer, mixed with 5 μL Annexin V + FITC and 5 μL PI, and incubated in the dark at room temperature for 15 min. Finally, the cells were mixed with 300 μL binding buffer. The experimental methods and analyses were reported in a previous study [30].

### Statistical analysis

The experimental data were expressed as mean ± standard deviation values. Statistical analysis was conducted by SPSS 21.0 software (SPSS Inc., Chicago, IL, USA). The unpaired Student’s t-test was used to compare differences between the two groups. A *P* value < 0.05 was considered to indicate a statistically significant difference. One-way analysis of variance for multiple-group comparisons was performed using GraphPad Prism 8 (GraphPad Software, San Diego, USA). A *P* value < 0.05 was considered statistically significant.

## Supplementary Information

**Additional file 1.**

**Additional file 2.**

## Data Availability

All of the data generated during this study are included in this article.

## Abbreviations

BPD: Bronchopulmonary dysplasia
Wnt: Wingless-related integration site
IHC: Immunohistochemistry
Dvl-1: Dishevelled-1
CCK-8: Cell Counting Kit-8
AECII: Alveolar type II epithelial cells
IQGAP1: IQ-domain GTPase-activating protein 1
Dvl-2: Dishevelled-2
PPARγ: Peroxysome proliferator activated receptor gamma
HRP: Horseradish peroxidase-conjugated
PVDF: Polyvinylidene difluoride
MDA: Malondialdehyde
SOD: Superoxide dismutase
RPMI: Roswell Park Memorial Institute
FITC: AnnexinV-fluorescein isothiocyanate
PI: Propidium iodide
SD: Sprague Dawley
PFA: Paraformaldehyde
RIPA: Radio Immunoprecipitation Assay
BCA: Quantified with Bicinchoninic Acid Assay
DAB: 3,3′-diaminobenzidine

## References

[1] Principi N, Pietro GMD, Esposito S (2018). Bronchopulmonary dysplasia: clinical aspects and preventive and therapeutic strategies. J Transl Med.

[2] Tracy MK, Berkelhamer SK (2019). Bronchopulmonary dysplasia and pulmonary outcomes of prematurity. Pediatr Ann.

[3] Willis KA, Weems MF (2019). Hemodynamically significant patent ductus arteriosus and the development of bronchopulmonary dysplasia. Congenit Heart Dis.

[4] Lohmann P, Luna RA, Hollister EB, Devaraj S, Mistretta T, Welty SE, Versalovic J (2014). The airway microbiome of intubated premature infants: characteristics and changes that predict the development of bronchopulmonary dysplasia. Pediatr Res.

[5] Chen LL, Zmuda EJ, Talavera MM, Frick J, Brock GN, Liu Y, Klebanoff MA, Trittmann JK (2020). Dual-specificity phosphatase (DUSP) genetic variants predict pulmonary hypertension in patients with bronchopulmonary dysplasia. Pediatr Res.

[6] Brugniaux JV, Coombs GB, Barak OF, Dujic Z, Sekhon MS, Ainslie PN (2018). Highs and lows of hyperoxia: physiological, performance, and clinical aspects. Am J Physiol Regul Integr Comp Physiol.

[7] Damiani E, Donati A, Girardis M (2018). Oxygen in the critically ill: friend or foe. Curr Opin Anaesthesiol.

[8] Smit B, Smulders YM, van der Wouden JC, Straaten HMO, Man AMES (2018). Hemodynamic effects of acute hyperoxia: systematic review and meta-analysis. Crit Care.

[9] Nova Z, Skovierova H, Calkovska A (2019). Alveolar-capillary membrane-related pulmonary cells as a target in endotoxin-induced acute lung injury. Int J Mol Sci.

[10] Moriyama G, Tanigawa M, Sakai K, Hirata Y, Kikuchi S, Saito Y, Kyoyama H, Matsuda K, Seike M, Gemma A (2018). Synergistic effect of targeting dishevelled-3 and the epidermal growth factor receptor-tyrosine kinase inhibitor on mesothelioma cells in vitro. Oncol Lett.

[11] Wang DP, Gu LL, Xue Q, Chen H, Mao GX (2018). CtBP2 promotes proliferation and reduces drug sensitivity in non-small cell lung cancer via the Wnt/β-catenin pathway. Neoplasma..

[12] Li X, Zhang T, Lv W, Wang H, Chen H, Xu Q, Cai H, Dai J (2022). Intratracheal administration of polystyrene microplastics induces pulmonary fibrosis by activating oxidative stress and Wnt/β-catenin signalling pathway in mice. Ecotoxicol Environ Saf.

[13] Zou W, Wang X, Sun R, Hu J, Ye D, Bai G, Liu S, Hong W, Guo M, Ran P (2021). PM2.5 induces airway remodeling in chronic obstructive pulmonary diseases via the Wnt 5a/β-catenin pathway. Int J Chron Obstruct Pulmon Dis.

[14] Guan H, Zhu T, Wu S, Liu S, Liu B, Wu J, Cai J, Zhu X, Zhang X, Zeng M (2019). Long noncoding RNA LINC00673-v4 promotes aggressiveness of lung adenocarcinoma via activating Wnt/β-catenin signaling. Proc Natl Acad Sci U S A.

[15] Das B, Sinha D (2019). Diallyl disulphide suppresses the cannonical Wnt signalling pathway and reverses the fibronectin-induced epithelial mesenchymal transition of A549 lung cancer cells. Food Funct.

[16] Tzouvelekis A, Gomatou G, Bouros E, Trigidou R, Tzilas V, Bouros D (2019). Common pathogenic mechanisms between idiopathic pulmonary fibrosis and lung cancer. Chest..

[17] Hwang SY, Deng X, Byun S, Lee C, Lee SJ, Suh H, Zhang J, Kang Q, Zhang T, Westover KD (2016). Direct targeting of β-catenin by a small molecule stimulates proteasomal degradation and suppresses oncogenic Wnt/β-catenin signaling. Cell Rep.

[18] Panfoli I, Candiano G, Malova M, Angelis LD, Cardiello V, Buonocore G, Ramenghi LA (2018). Oxidative stress as a primary risk factor for brain damage in preterm newborns. Front Pediatr.

[19] Batlahally S, Franklin A, Damianos A, Huang J, Chen P, Sharma M, Duara J, Keerthy D, Zambrano R, Shehadeh LA (2020). Soluble klotho, a biomarker and therapeutic strategy to reduce bronchopulmonary dysplasia and pulmonary hypertension in preterm infants. Sci Rep.

[20] Lee YN, Gao Y, Wang HY (2008). Differential mediation of the Wnt canonical pathway by mammalian Dishevelleds-1, −2, and −3. Cell Signal.

[21] Zhao H, Xie C, Lin X, Zhao Y, Han Y, Fan C, Zhang X, Du J, Han Y, Han Q (2014). Coexpression of IQ-domain GTPase-activating protein 1 (IQGAP1) and Dishevelled (DVL-1) is correlated with poor prognosis in non-small cell lung cancer. PLoS One.

[22] Jin C, Song P, Pang J (2019). The CK2 inhibitor CX4945 reverses cisplatin resistance in the A549/DDP human lung adenocarcinoma cell line. Oncol Lett.

[23] Li Y, Sheng H, Ma F, Wu Q, Huang J, Chen Q, Sheng L, Zhu X, Zhu X, Xu M (2021). RNA m(6)A reader YTHDF2 facilitates lung adenocarcinoma cell proliferation and metastasis by targeting the AXIN1/Wnt/β-catenin signaling. Cell Death Dis.

[24] Sucre JMS, Deutsch GH, Jetter CS, Ambalavanan N, Benjamin JT, Gleaves LA, Millis BA, Young LR, Blackwell TS, Kropski JA (2018). A shared pattern of β-catenin activation in bronchopulmonary dysplasia and idiopathic pulmonary fibrosis. Am J Pathol.

[25] Lecarpentier Y, Gourrier E, Gobert V, Vallée A (2019). Bronchopulmonary dysplasia: crosstalk between PPARɣ, Wnt/β-catenin and TGF-β pathways; the potential therapeutic role of PPARɣ agonists. Front Pediatr.

[26] Wu D, Liang M, Dang H, Fang F, Xu F, Liu C (2018). Hydrogen protects against hyperoxia-induced apoptosis in type II alveolar epithelial cells via activation of PI3K/Akt/Foxo3a signalling pathway. Biochem Biophys Res Commun.

[27] Huang B, Fu H, Yang M, Fang F, Kuang F, Xu F (2009). Neuropeptide substance P attenuates hyperoxia-induced oxidative stress injury in type II alveolar epithelial cells via suppressing the activation of JNK pathway. Lung..

[28] Jin H, Ciechanowicz AK, Kaplan AR, Wang L, Zhang PX, Lu YC, Tobin RE, Tobin BA, Cohn L, Zeiss CJ (2018). Surfactant protein C dampens inflammation by decreasing JAK/STAT activation during lung repair. Am J Physiol Lung Cell Mol Physiol.

[29] Chen Y, Xia R, Huang Y (2016). An immunostimulatory dual-functional nanocarrier that improves cancer immunochemotherapy. Nat Commun.

[30] Bento CF, Ashkenazi A, Jimenez-Sanchez M (2016). The Parkinson's disease-associated genes ATP13A2 and SYT11 regulate autophagy via a common pathway. Nat Commun.

