# Supplementary material for: Hyperoxia exposure upregulates Dvl-1 and activates Wnt/β-catenin signaling pathway in newborn rat lung
Source: BMC Mol Cell Biol. 2023 Feb 2;24:4. doi: 10.1186/s12860-023-00465-6 (PMC9893620; doi:10.1186/s12860-023-00465-6)
Supplement: Supplementary file 1 — Additional file 1. [file 12860_2023_465_MOESM1_ESM.pdf]

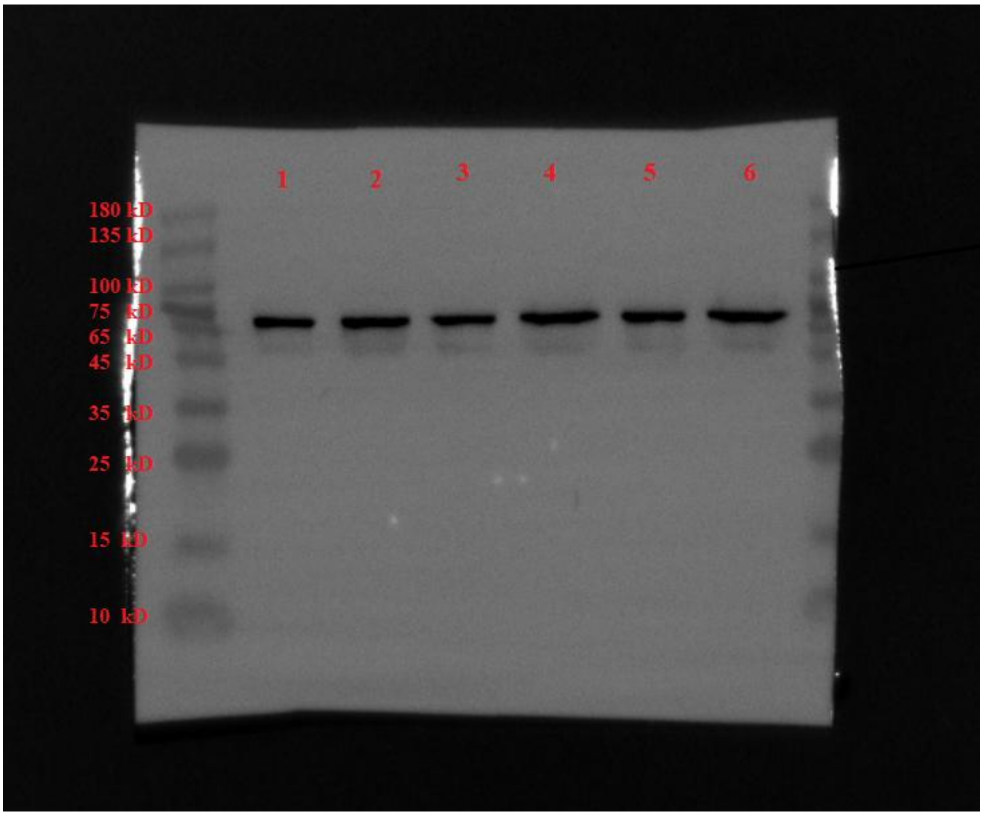

CTNNBL1-uncropped-replicate-1

1: normoxia exposure on 3rd day, **2:** hyporoxia exposure on 3rd day, **3:** normoxia exposure on 7th day, **4:** hyporoxia exposure on 7th day, **5:** normoxia exposure on 14th day, **6:** hyporoxia exposure on 14th day.

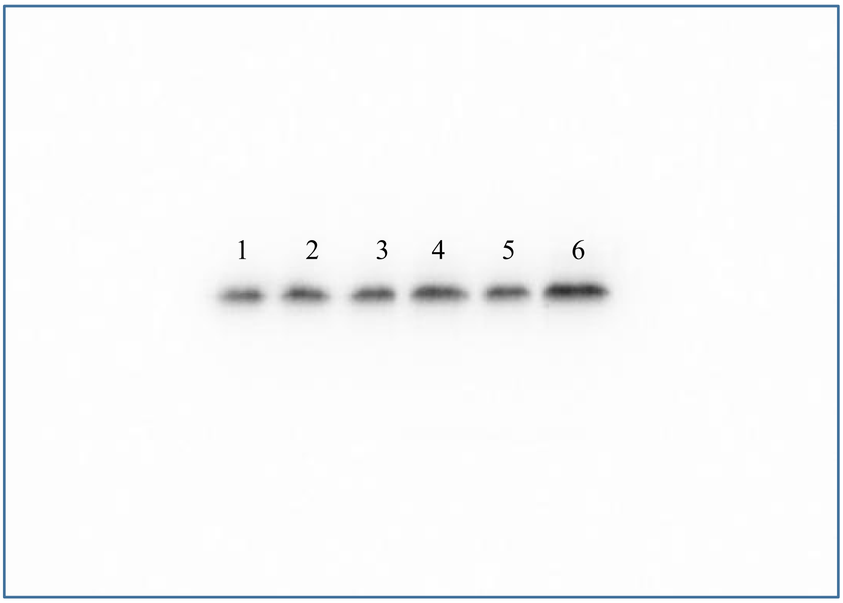

CTNNBL1 replicate-2

1: normoxia exposure on 3rd day, **2:** hyporoxia exposure on 3rd day, **3:** normoxia exposure on 7th day, **4:** hyporoxia exposure on 7th day, **5:** normoxia exposure on 14th day, **6:** hyporoxia exposure on 14th day.

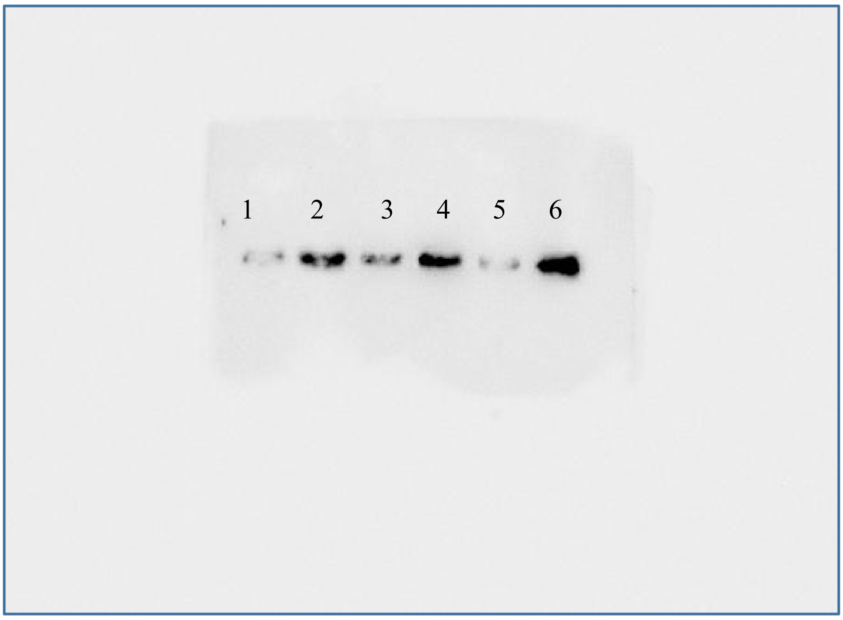

CTNNBL1 replicate-3

1: normoxia exposure on 3rd day, **2:** hyporoxia exposure on 3rd day, **3:** normoxia exposure on 7th day, **4:** hyporoxia exposure on 7th day, **5:** normoxia exposure on 14th day, **6:** hyporoxia exposure on 14th day.

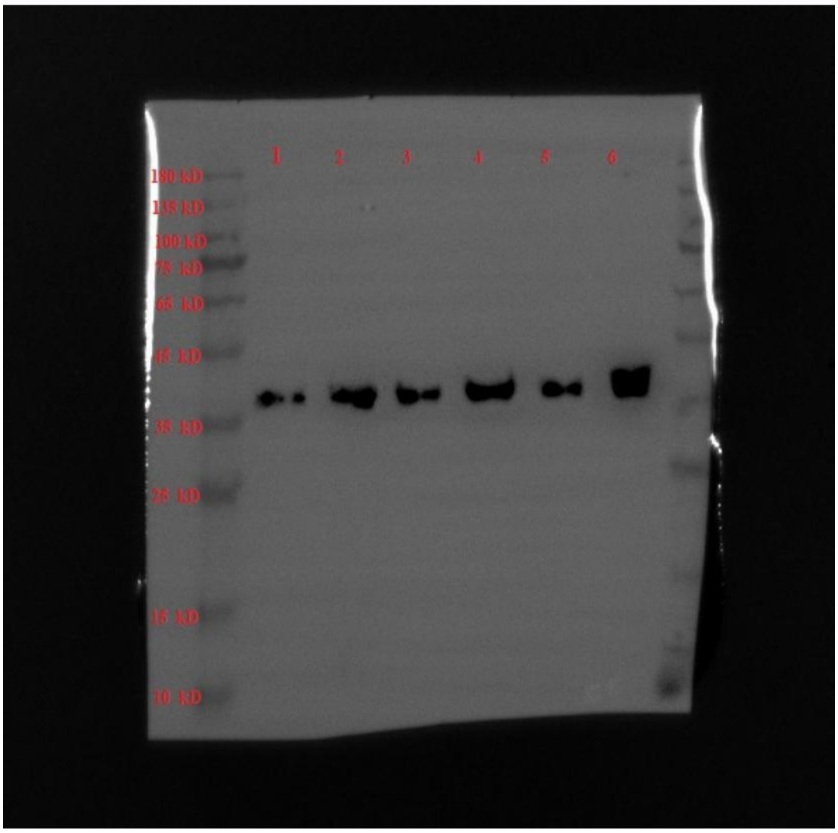

CYCLIND1-uncropped-replicate-1

1: normoxia exposure on 3rd day, **2:** hyporoxia exposure on 3rd day, **3:** normoxia exposure on 7th day, **4:** hyporoxia exposure on 7th day, **5:** normoxia exposure on 14th day, **6:** hyporoxia exposure on 14th day.

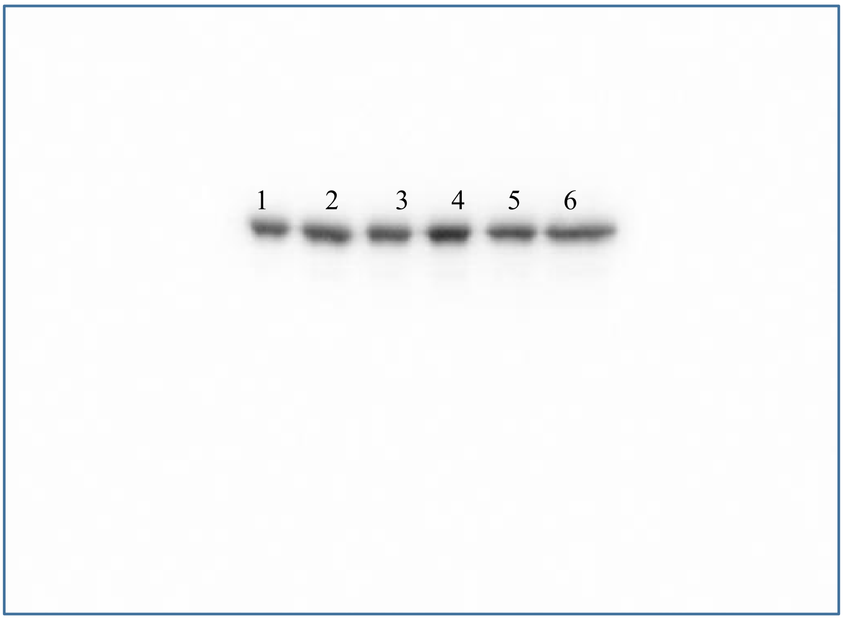

CYCLIND1-replicate-2

1: normoxia exposure on 3rd day, **2:** hyporoxia exposure on 3rd day, **3:** normoxia exposure on 7th day, **4:** hyporoxia exposure on 7th day, **5:** normoxia exposure on 14th day, **6:** hyporoxia exposure on 14th day.

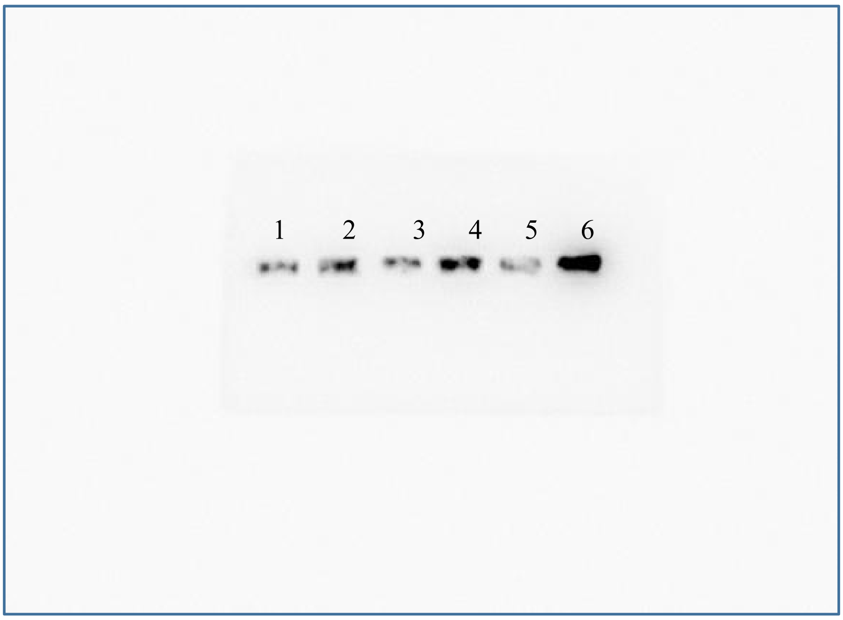

CYCLIND1-replicate-3

1: normoxia exposure on 3rd day, **2:** hyporoxia exposure on 3rd day, **3:** normoxia exposure on 7th day, **4:** hyporoxia exposure on 7th day, **5:** normoxia exposure on 14th day, **6:** hyporoxia exposure on 14th day.

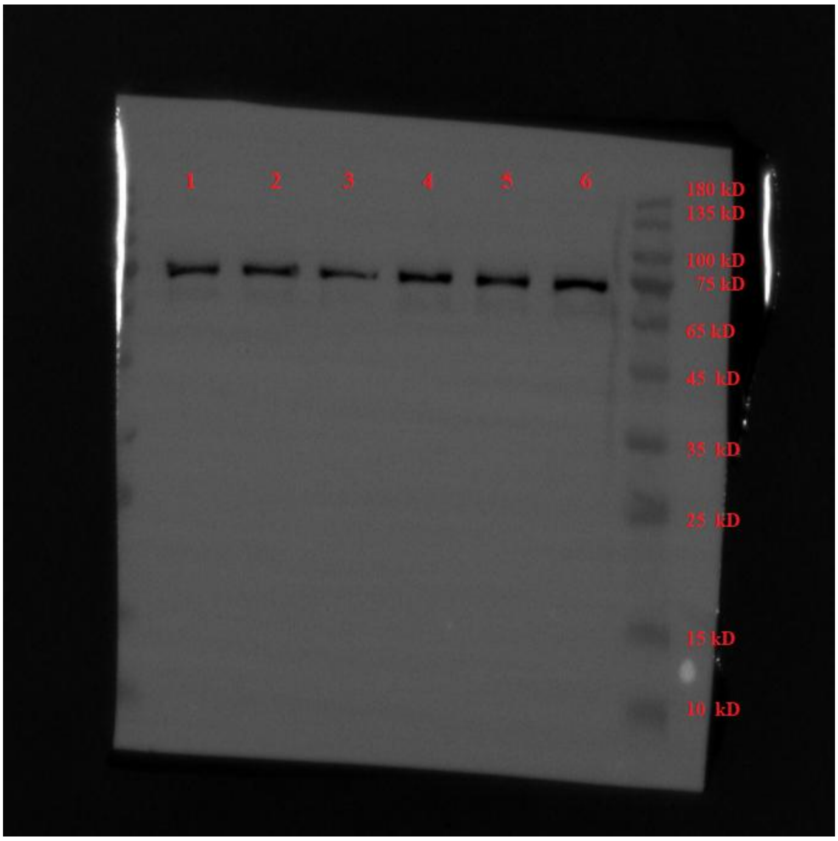

DVL-1-uncropped-replicate-1

1: normoxia exposure on 3rd day, **2:** hyporoxia exposure on 3rd day, **3:** normoxia exposure on 7th day, **4:** hyporoxia exposure on 7th day, **5:** normoxia exposure on 14th day, **6:** hyporoxia exposure on 14th day.

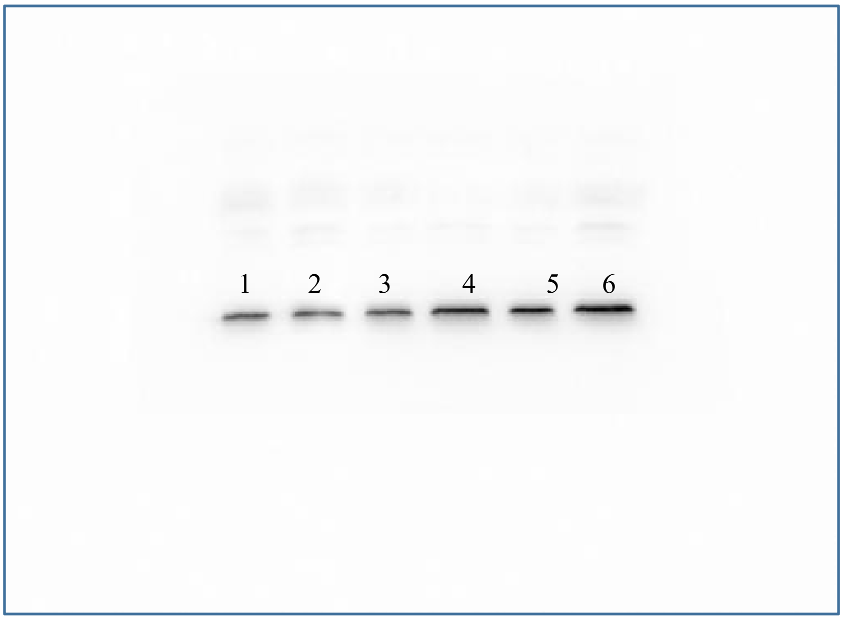

DVL-1 replicate-2

1: normoxia exposure on 3rd day, **2:** hyporoxia exposure on 3rd day, **3:** normoxia exposure on 7th day, **4:** hyporoxia exposure on 7th day, **5:** normoxia exposure on 14th day, **6:** hyporoxia exposure on 14th day.

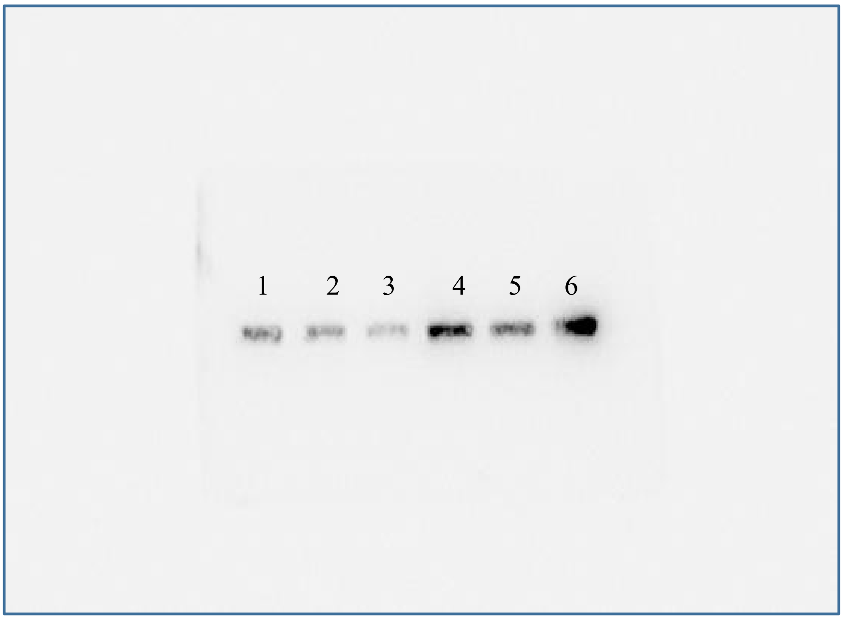

DVL-1 replicate-3

1: normoxia exposure on 3rd day, **2:** hyporoxia exposure on 3rd day, **3:** normoxia exposure on 7th day, **4:** hyporoxia exposure on 7th day, **5:** normoxia exposure on 14th day, **6:** hyporoxia exposure on 14th day.

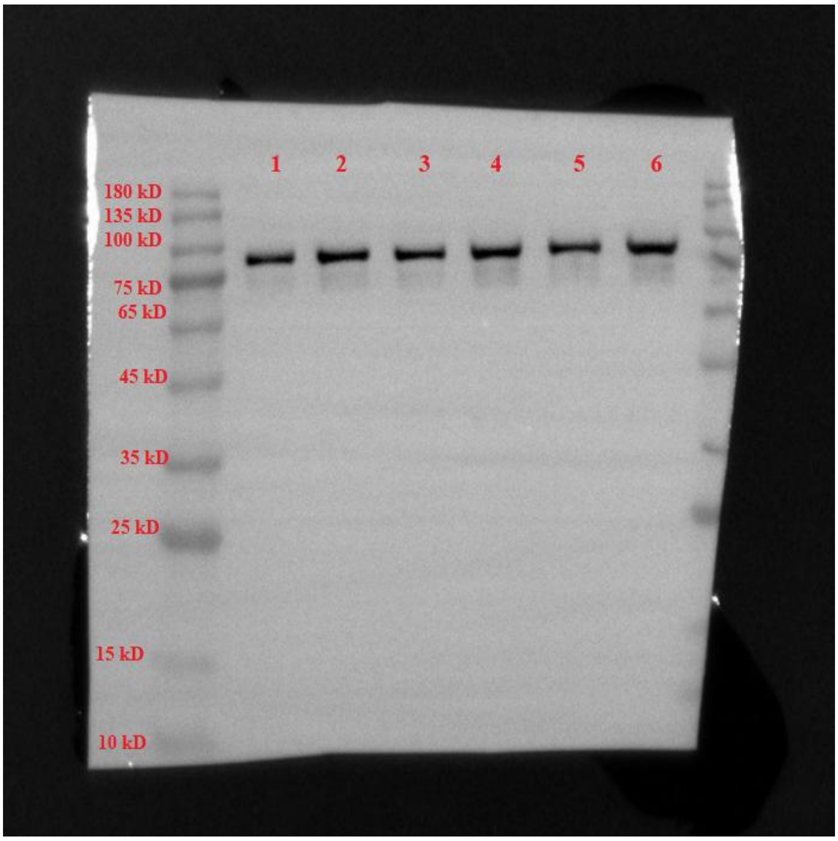

β-CATENIN-uncropped-replicate-1

1: normoxia exposure on 3rd day, **2:** hyporoxia exposure on 3rd day, **3:** normoxia exposure on 7th day, **4:** hyporoxia exposure on 7th day, **5:** normoxia exposure on 14th day, **6:** hyporoxia exposure on 14th day.

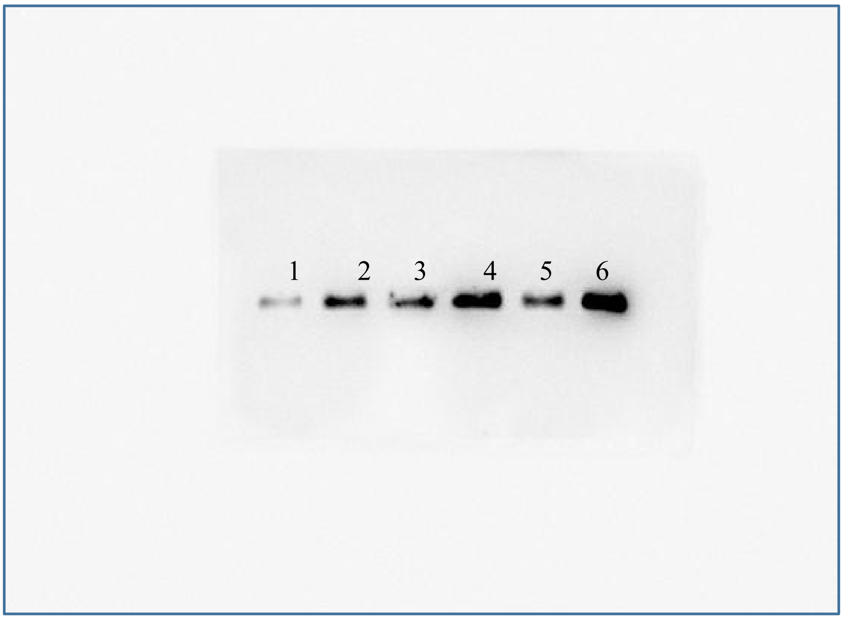

β-CATENIN-replicate-2

1: normoxia exposure on 3rd day, **2:** hyporoxia exposure on 3rd day, **3:** normoxia exposure on 7th day, **4:** hyporoxia exposure on 7th day, **5:** normoxia exposure on 14th day, **6:** hyporoxia exposure on 14th day.

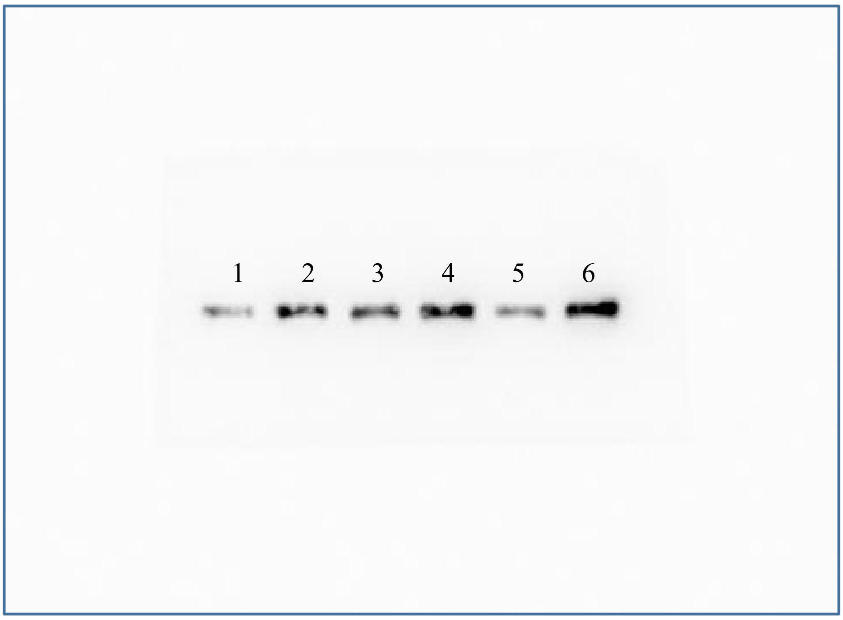

β-CATENIN-replicate-3

1: normoxia exposure on 3rd day, **2:** hyporoxia exposure on 3rd day, **3:** normoxia exposure on 7th day, **4:** hyporoxia exposure on 7th day, **5:** normoxia exposure on 14th day, **6:** hyporoxia exposure on 14th day.

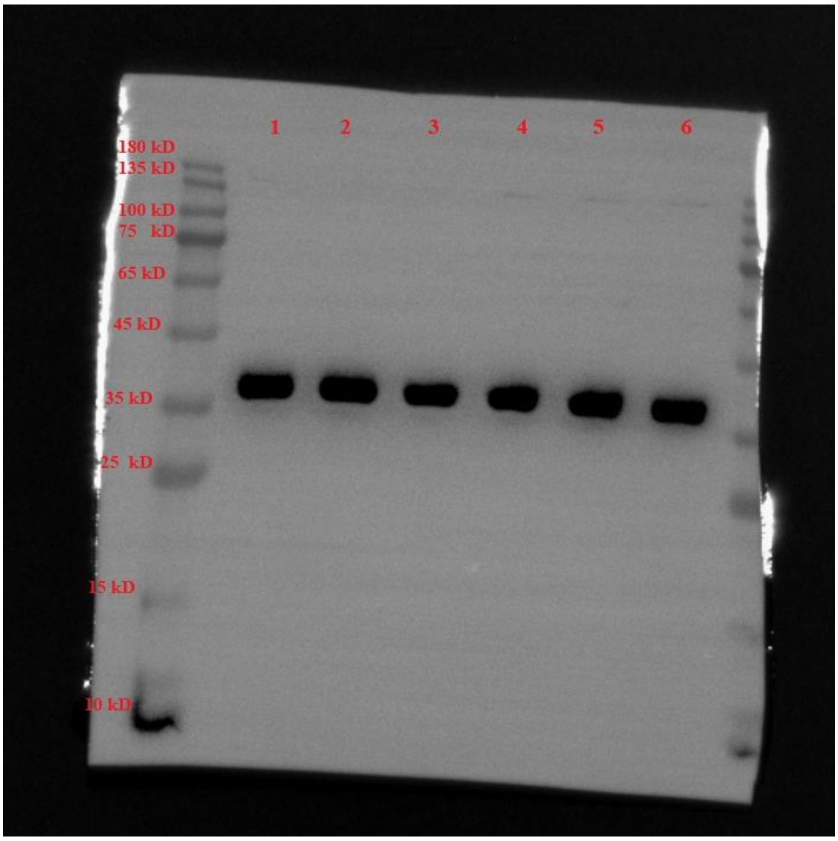

GAPDH-uncropped-replicate-1

1: normoxia exposure on 3rd day, **2:** hyporoxia exposure on 3rd day, **3:** normoxia exposure on 7th day, **4:** hyporoxia exposure on 7th day, **5:** normoxia exposure on 14th day, **6:** hyporoxia exposure on 14th day.

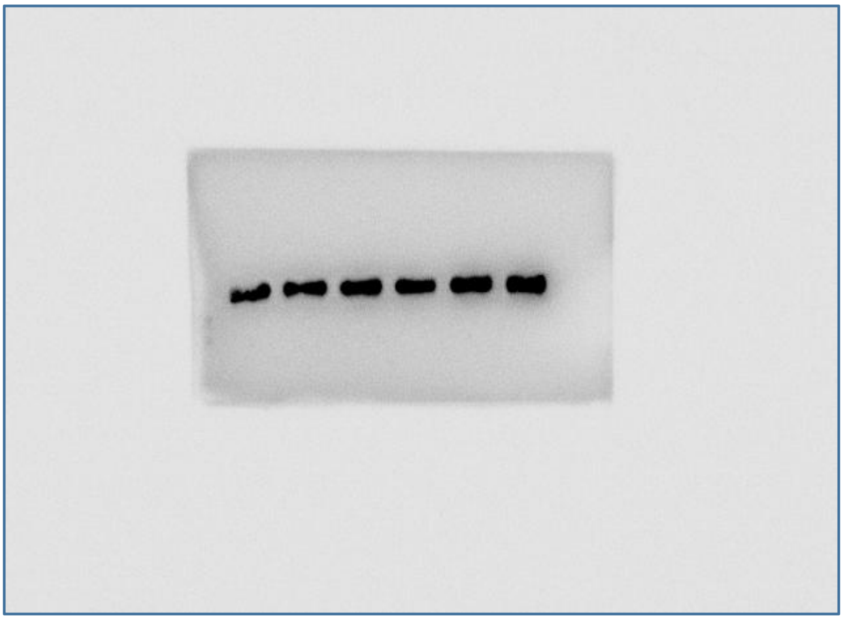

GAPDH-replicate-2

1: normoxia exposure on 3rd day, **2:** hyporoxia exposure on 3rd day, **3:** normoxia exposure on 7th day, **4:** hyporoxia exposure on 7th day, **5:** normoxia exposure on 14th day, **6:** hyporoxia exposure on 14th day.

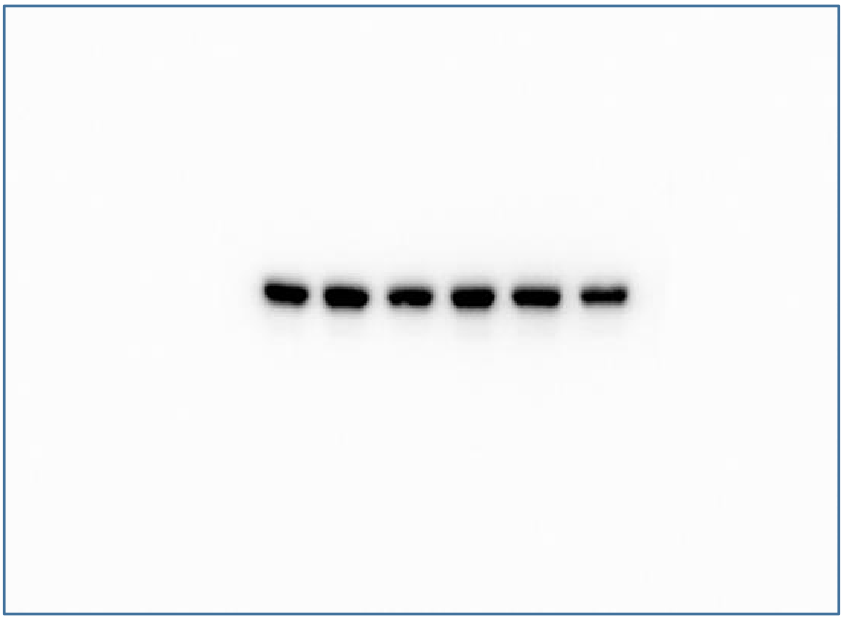

GAPDH-replicate-3

1: normoxia exposure on 3rd day, **2:** hyporoxia exposure on 3rd day, **3:** normoxia exposure on 7th day, **4:** hyporoxia exposure on 7th day, **5:** normoxia exposure on 14th day, **6:** hyporoxia exposure on 14th day.
