# Supplementary material for: Hyperoxia exposure upregulates Dvl-1 and activates Wnt/β-catenin signaling pathway in newborn rat lung
Source: BMC Mol Cell Biol. 2023 Feb 2;24:4. doi: 10.1186/s12860-023-00465-6 (PMC9893620; doi:10.1186/s12860-023-00465-6)
Supplement: Supplementary file 2 — Additional file 2. [file 12860_2023_465_MOESM2_ESM.pdf]

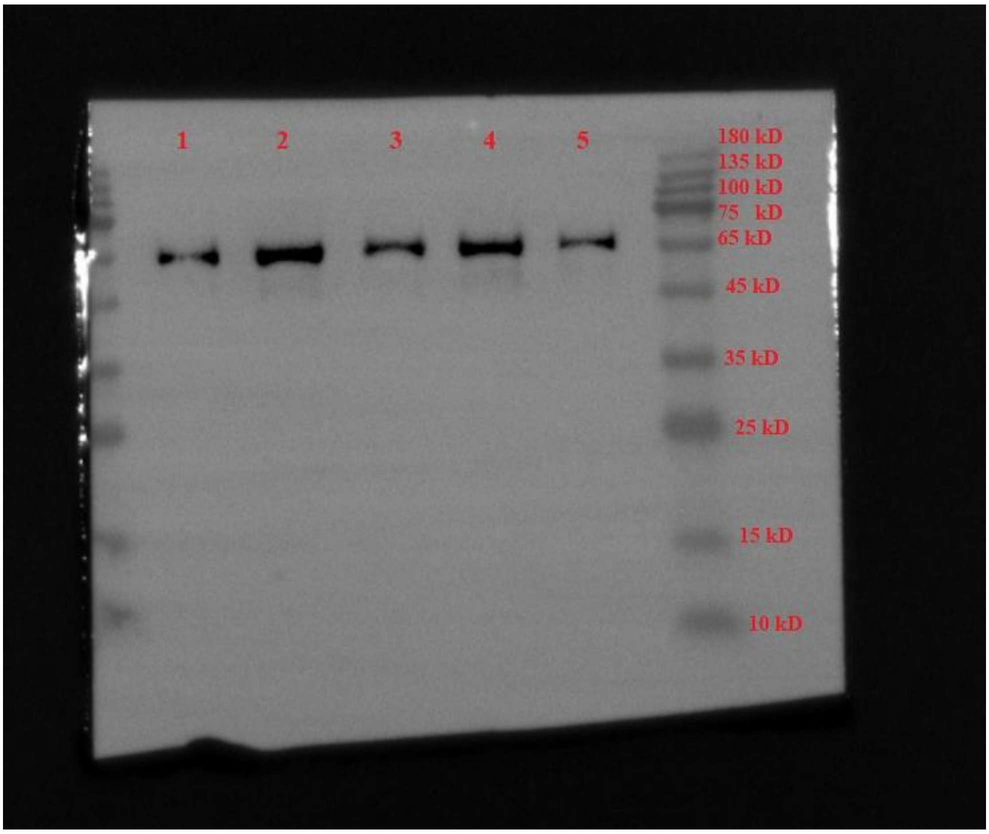

CTNNBL1 -uncropped -replicate-1

1: Control, **2:** Hyporoxia, 3: si-Dvl-1, 4: si-Dvl-1+ Hyporoxia, 5: si-Dvl-1+ Hyporoxia+ MSAB.

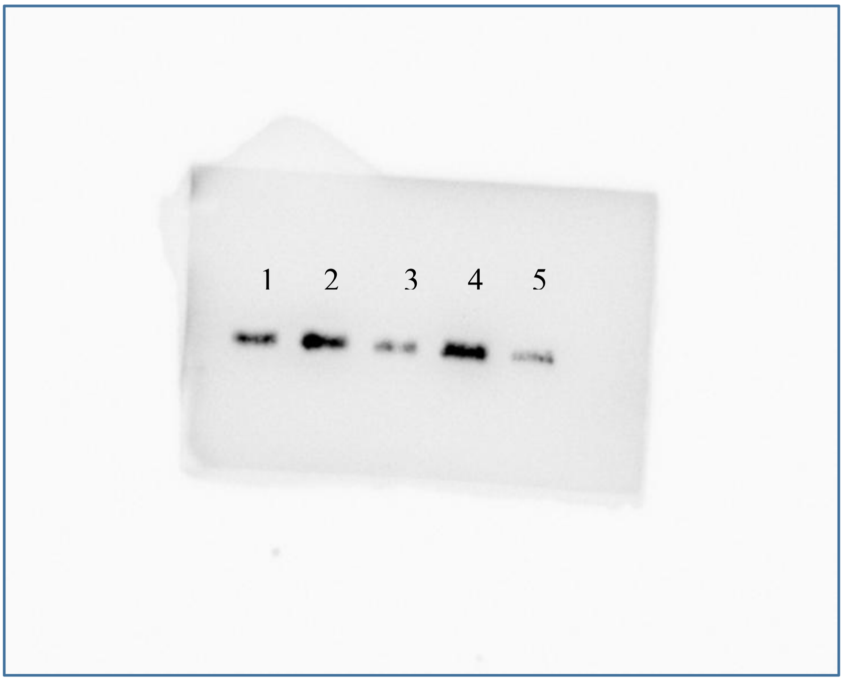

CTNNBL1 -replicate-2

1: Control, **2:** Hyporoxia, 3: si-Dvl-1, 4: si-Dvl-1+ Hyporoxia, 5: si-Dvl-1+ Hyporoxia+ MSAB.

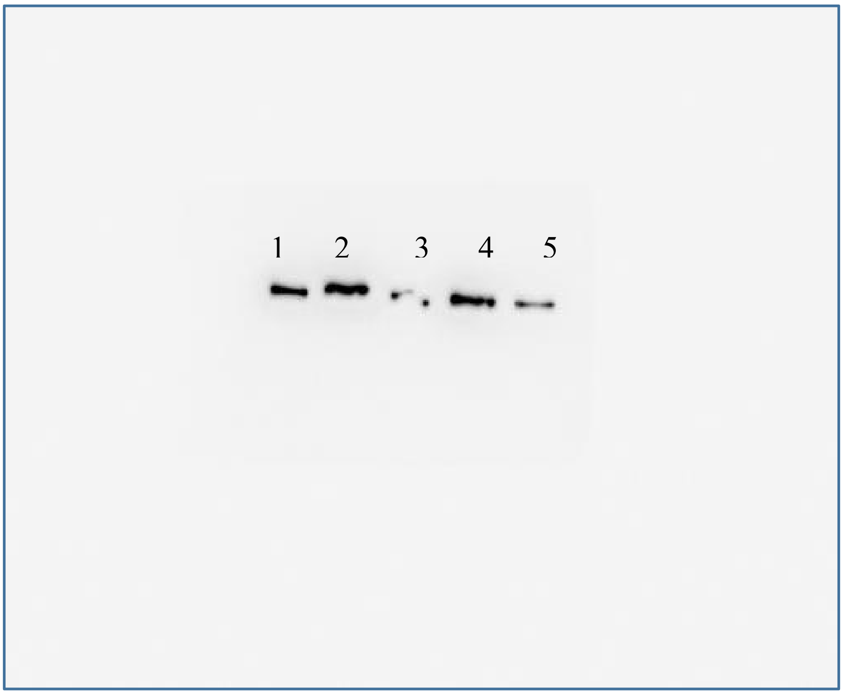

CTNNBL1 -replicate-3

1: Control, **2:** Hyporoxia, 3: si-Dvl-1, 4: si-Dvl-1+ Hyporoxia, 5: si-Dvl-1+ Hyporoxia+ MSAB.

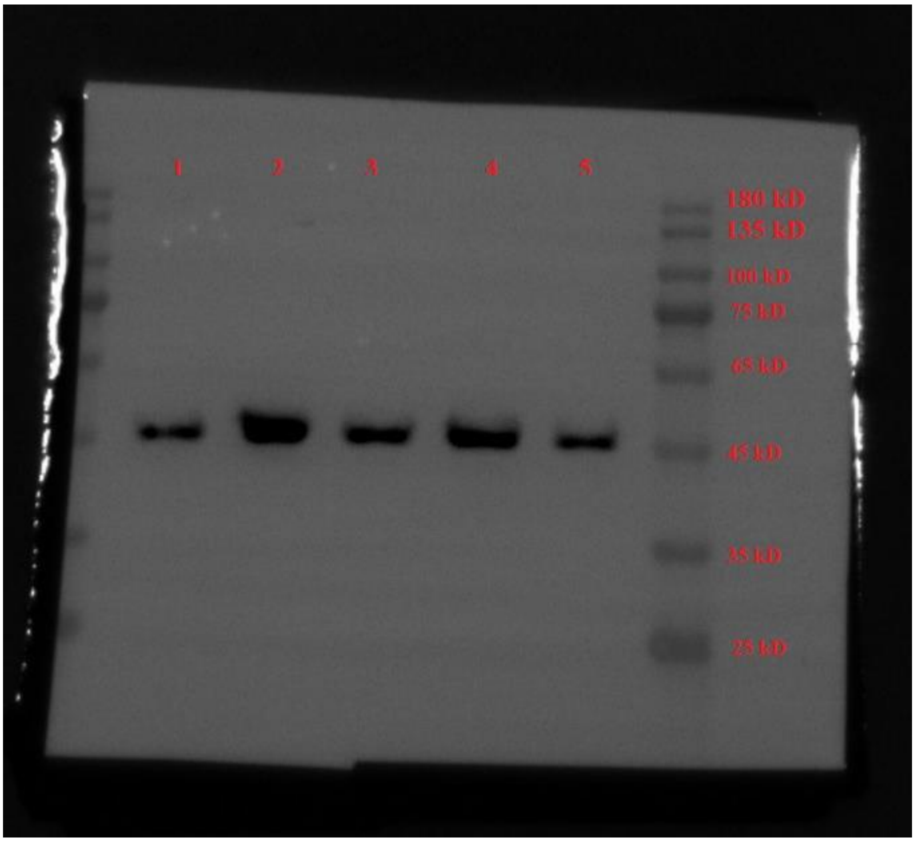

GSK-3β-uncropped -replicate-1

1: Control, **2:** Hyporoxia, 3: si-Dvl-1, 4: si-Dvl-1+ Hyporoxia, 5: si-Dvl-1+ Hyporoxia+ MSAB.

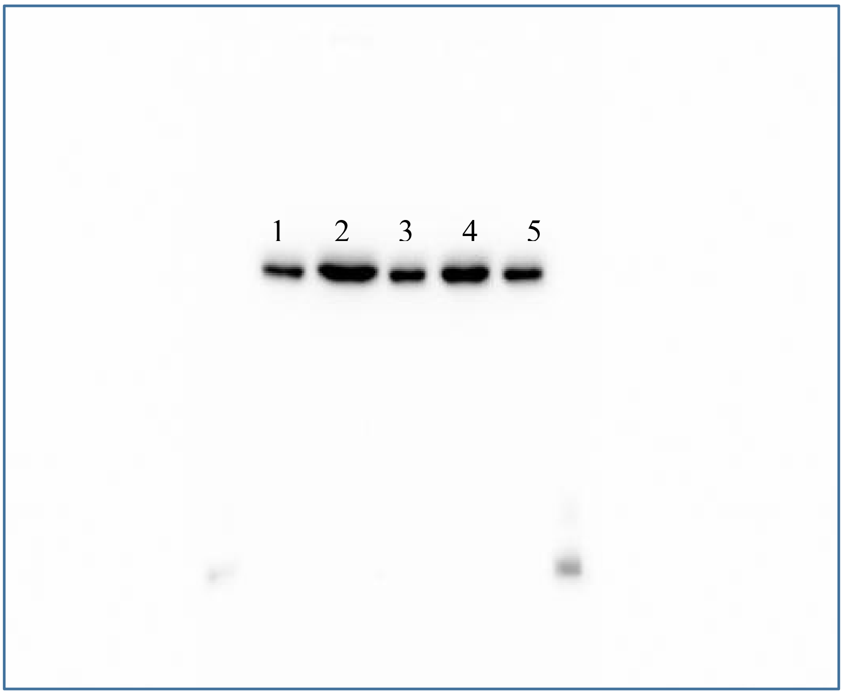

GSK-3β -replicate-2

1: Control, **2:** Hyporoxia, 3: si-Dvl-1, 4: si-Dvl-1+ Hyporoxia, 5: si-Dvl-1+ Hyporoxia+ MSAB.

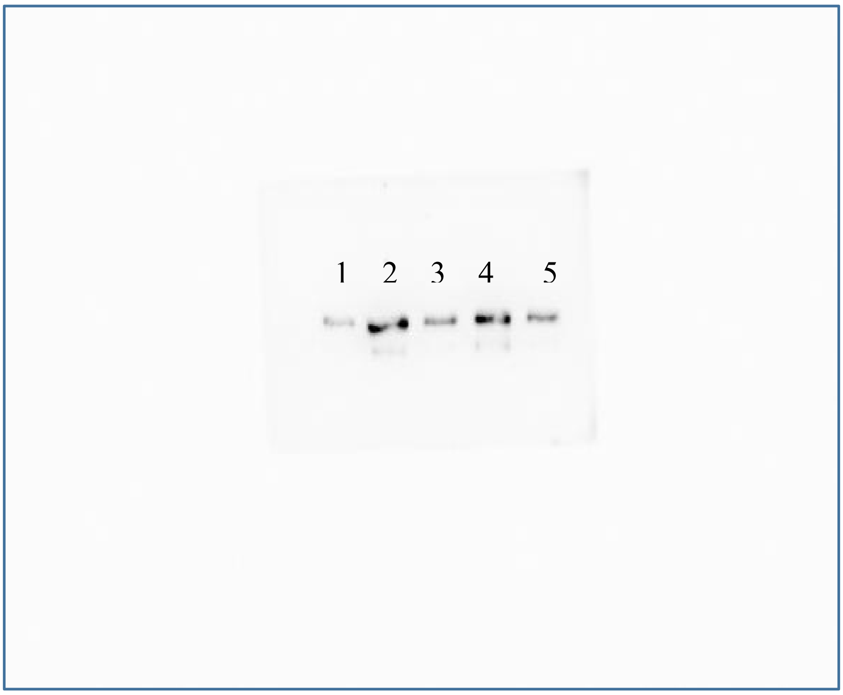

GSK-3β -replicate-3

1: Control, **2:** Hyporoxia, 3: si-Dvl-1, 4: si-Dvl-1+ Hyporoxia, 5: si-Dvl-1+ Hyporoxia+ MSAB.

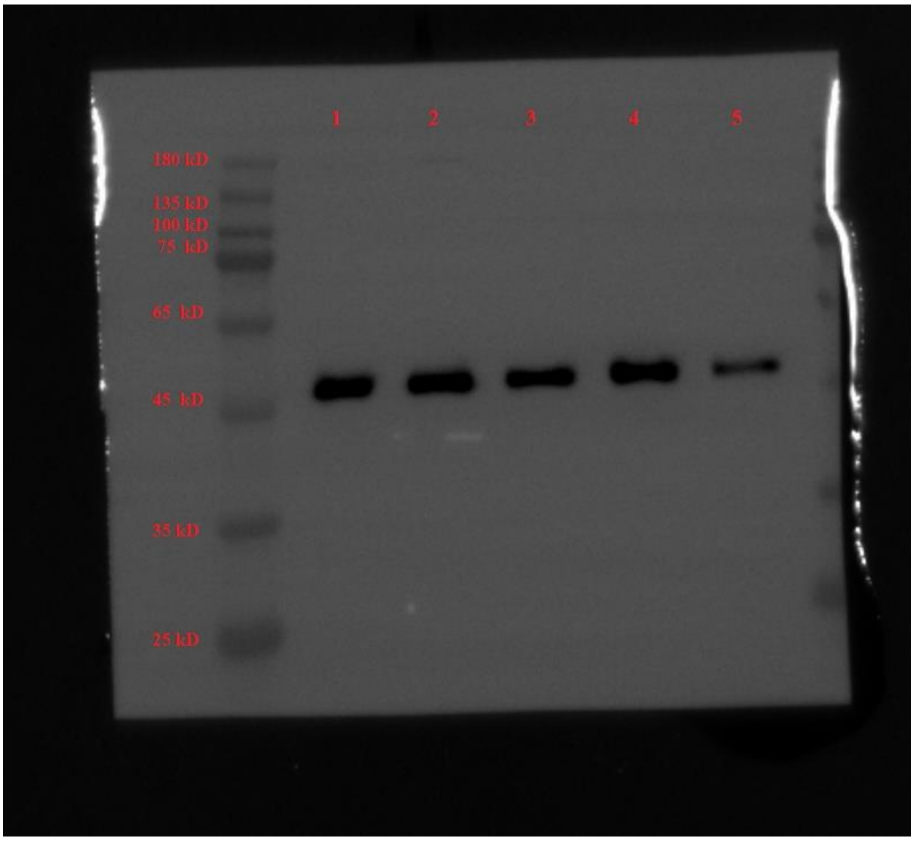

p-GSK-3β-uncropped -replicate-1

1: Control, 2: Hyporoxia, 3: si-Dvl-1, 4: si-Dvl-1+ Hyporoxia, 5: si-Dvl-1+ Hyporoxia+ MSAB.

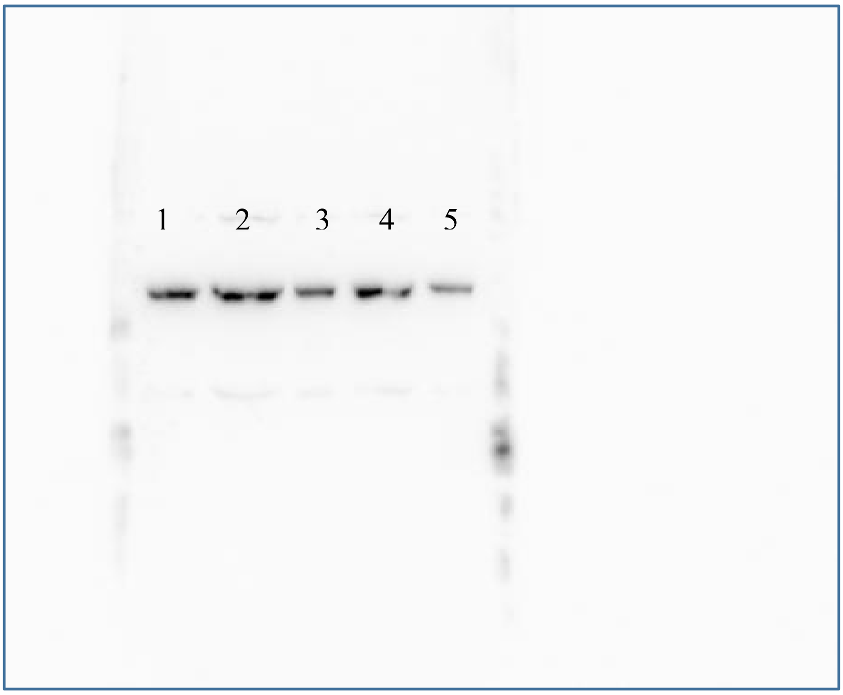

p-GSK-3β-uncropped -replicate-2

1: Control, 2: Hyporoxia, 3: si-Dvl-1, 4: si-Dvl-1+ Hyporoxia, 5: si-Dvl-1+ Hyporoxia+ MSAB.

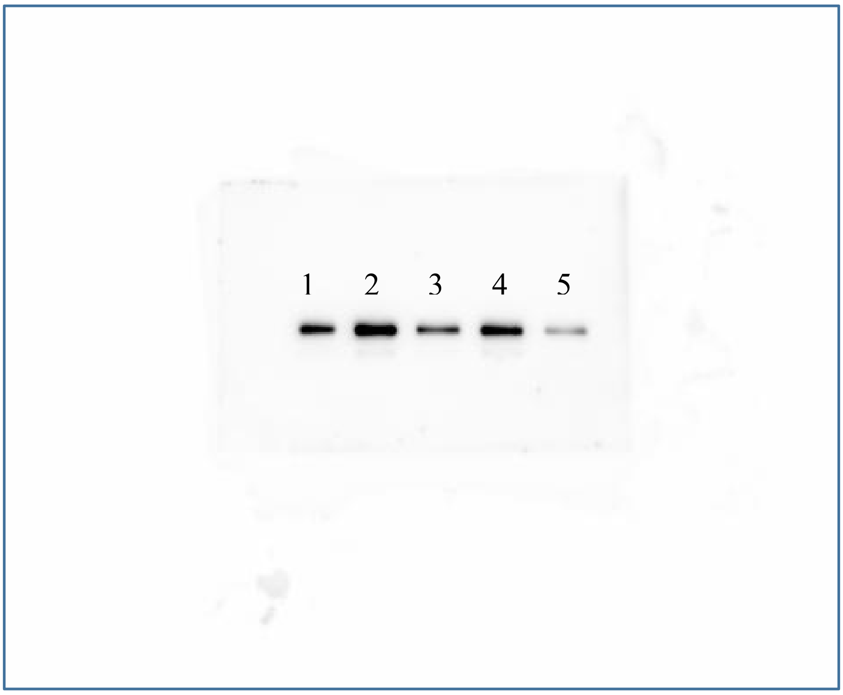

p-GSK-3β-uncropped -replicate-3

1: Control, 2: Hyporoxia, 3: si-Dvl-1, 4: si-Dvl-1+ Hyporoxia, 5: si-Dvl-1+ Hyporoxia+ MSAB.

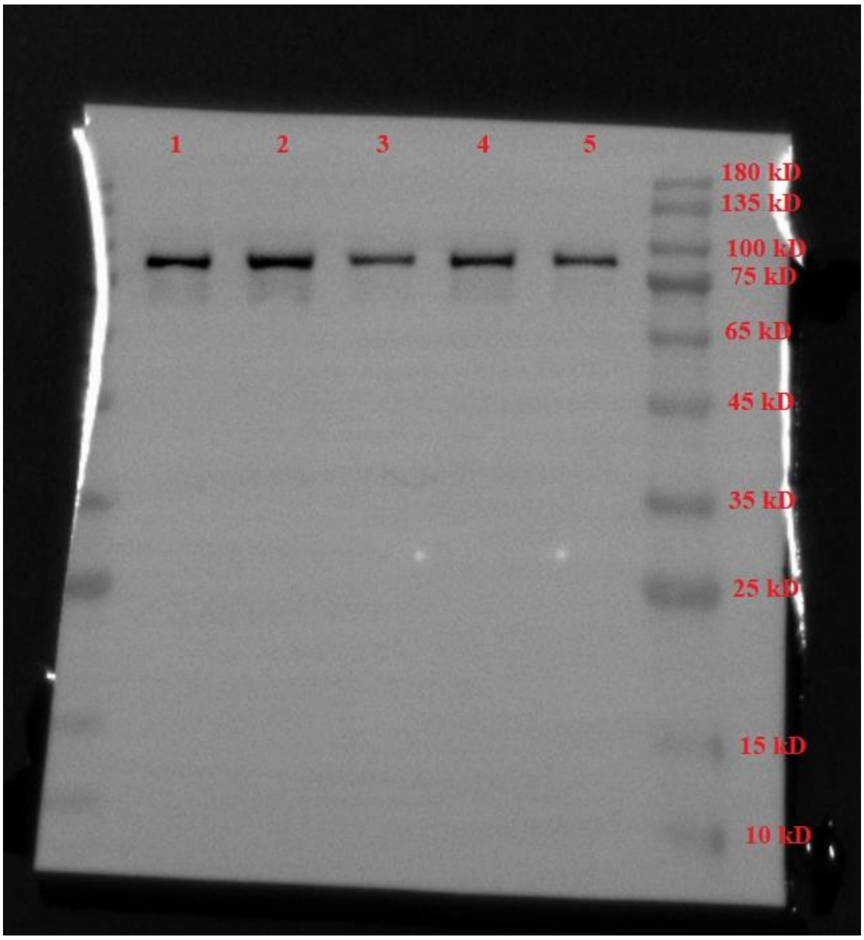

β-CATENIN -uncropped -replicate-1

1: Control, 2: Hyporoxia, 3: si-Dvl-1, 4: si-Dvl-1+ Hyporoxia, 5: si-Dvl-1+ Hyporoxia+ MSAB.

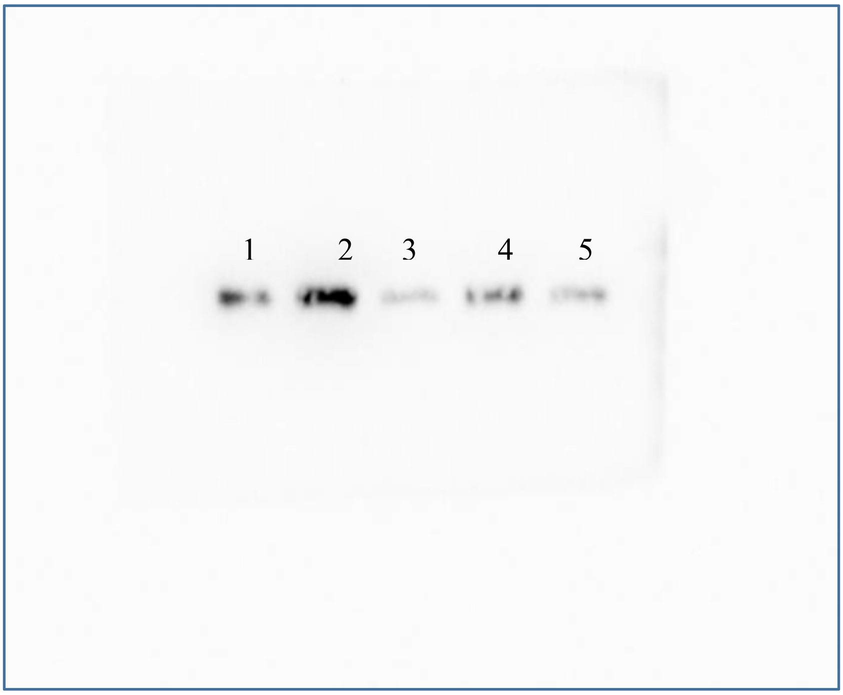

β-CATENIN -uncropped -replicate-2

1: Control, 2: Hyporoxia, 3: si-Dvl-1, 4: si-Dvl-1+ Hyporoxia, 5: si-Dvl-1+ Hyporoxia+ MSAB.

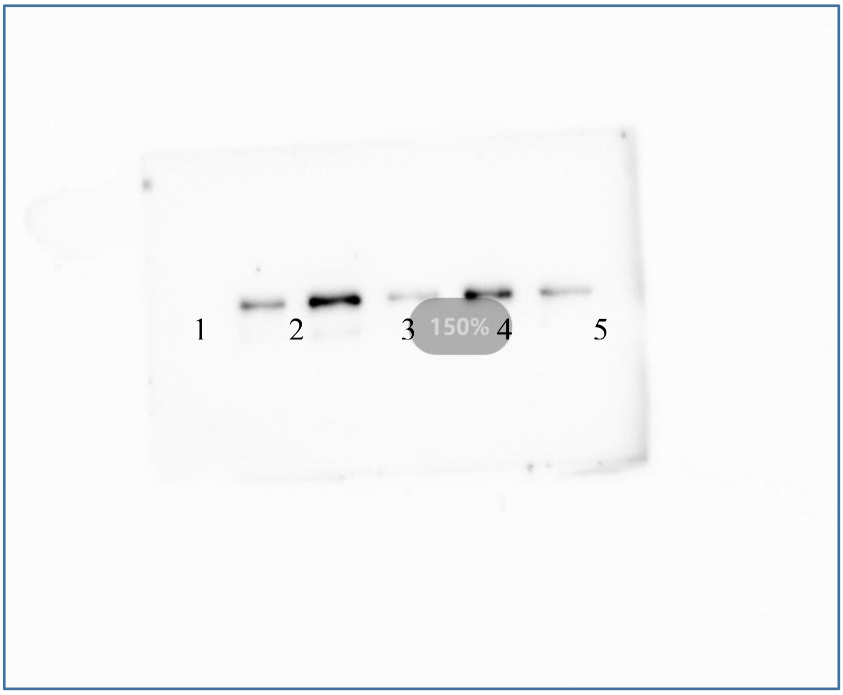

β-CATENIN -uncropped -replicate-3

1: Control, 2: Hyporoxia, 3: si-Dvl-1, 4: si-Dvl-1+ Hyporoxia, 5: si-Dvl-1+ Hyporoxia+ MSAB.

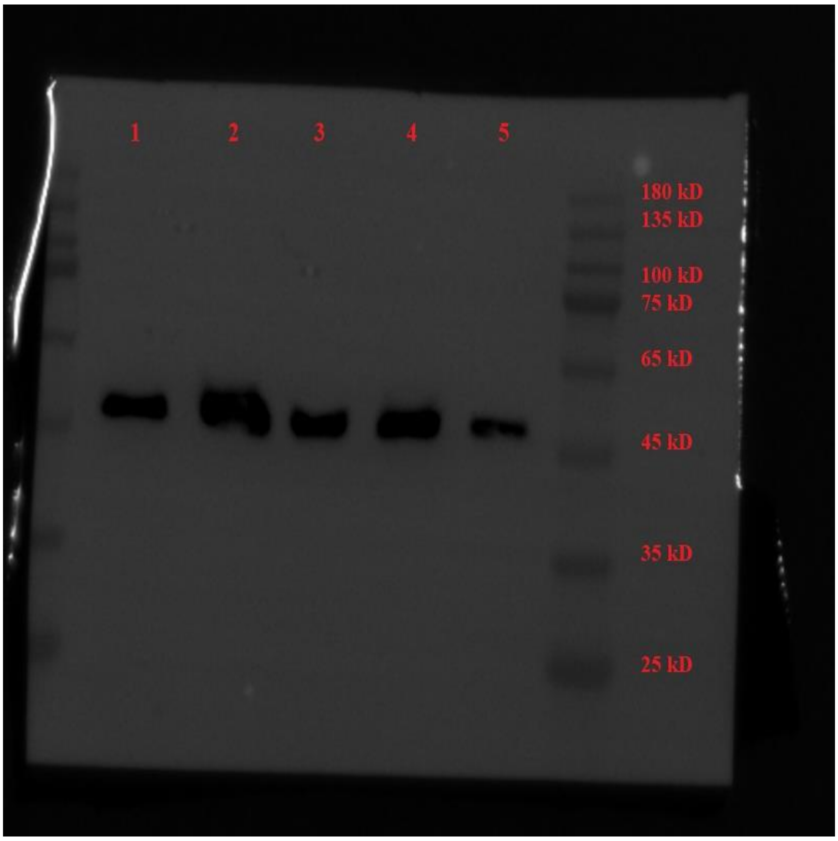

CYCLIND1-uncropped-replicate-1

1: Control, 2: Hyporoxia, 3: si-Dvl-1, 4: si-Dvl-1+ Hyporoxia, 5: si-Dvl-1+ Hyporoxia+ MSAB.

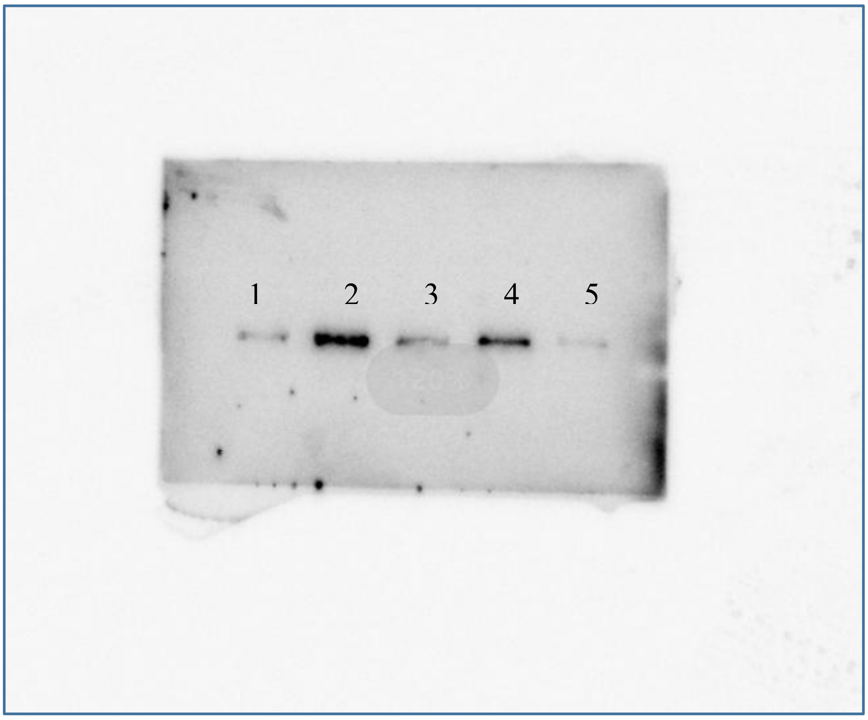

CYCLIND1-replicate-2

1: Control, 2: Hyporoxia, 3: si-Dvl-1, 4: si-Dvl-1+ Hyporoxia, 5: si-Dvl-1+ Hyporoxia+ MSAB.

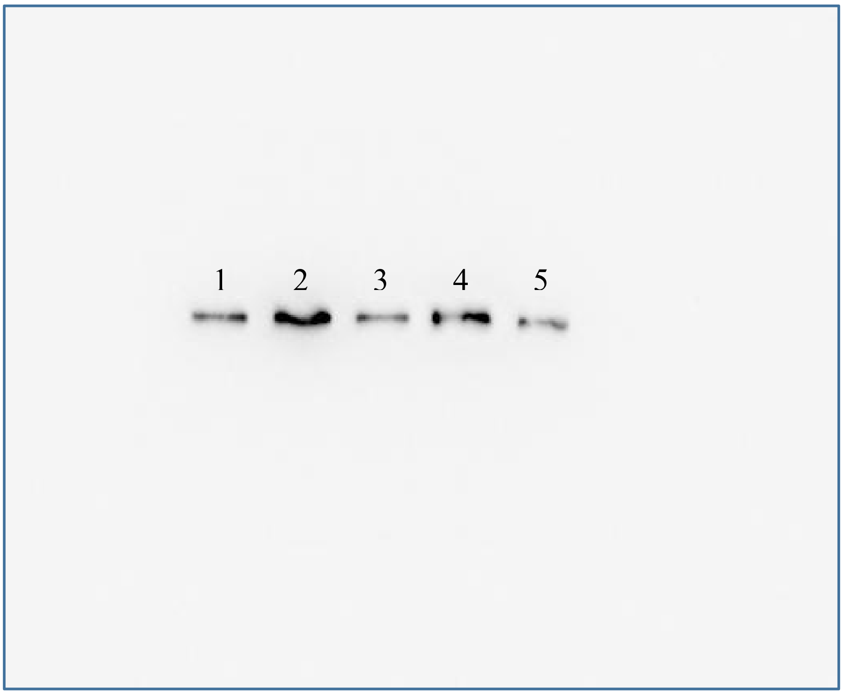

CYCLIND1-replicate-3

1: Control, 2: Hyporoxia, 3: si-Dvl-1, 4: si-Dvl-1+ Hyporoxia, 5: si-Dvl-1+ Hyporoxia+ MSAB.

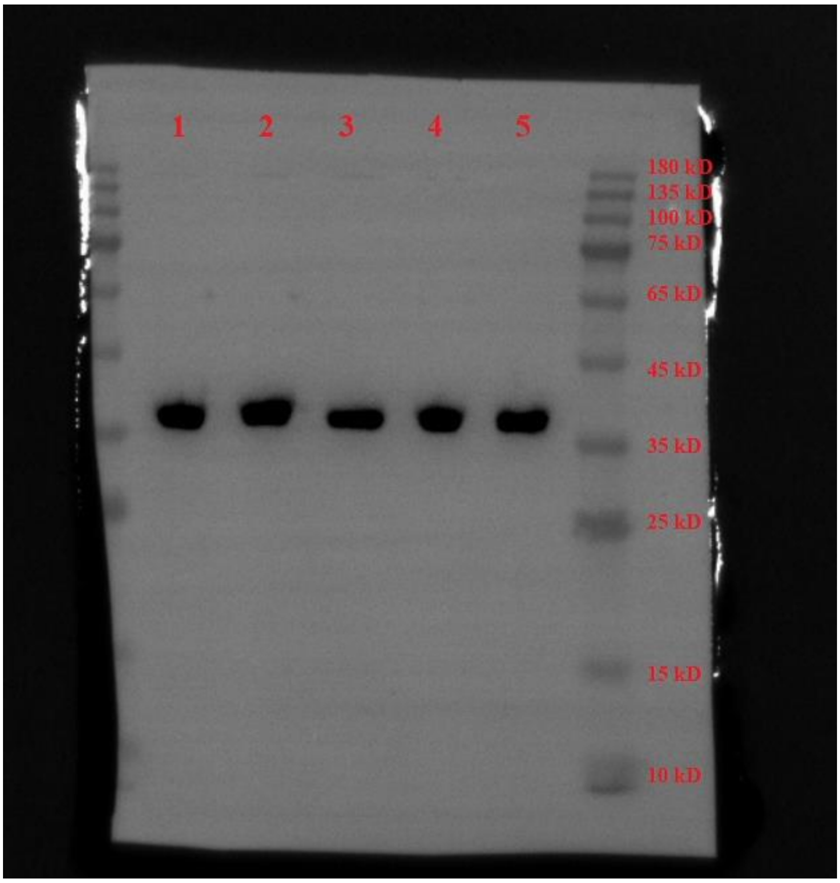

GAPDH-uncropped-replicate-1

1: Control, 2: Hyporoxia, 3: si-Dvl-1, 4: si-Dvl-1+ Hyporoxia, 5: si-Dvl-1+ Hyporoxia+ MSAB.

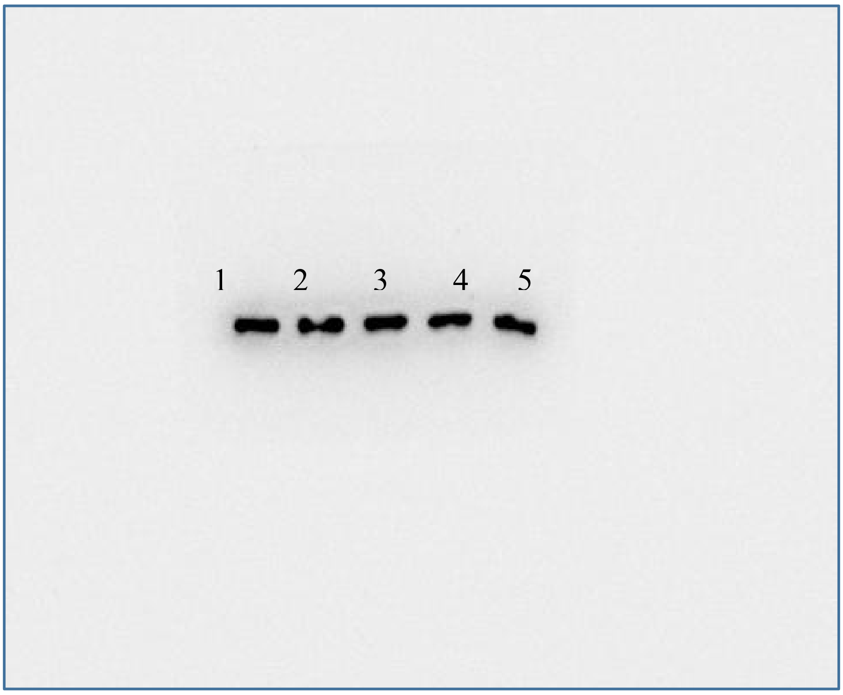

GAPDH-replicate-2

1: Control, 2: Hyporoxia, 3: si-Dvl-1, 4: si-Dvl-1+ Hyporoxia, 5: si-Dvl-1+ Hyporoxia+ MSAB.

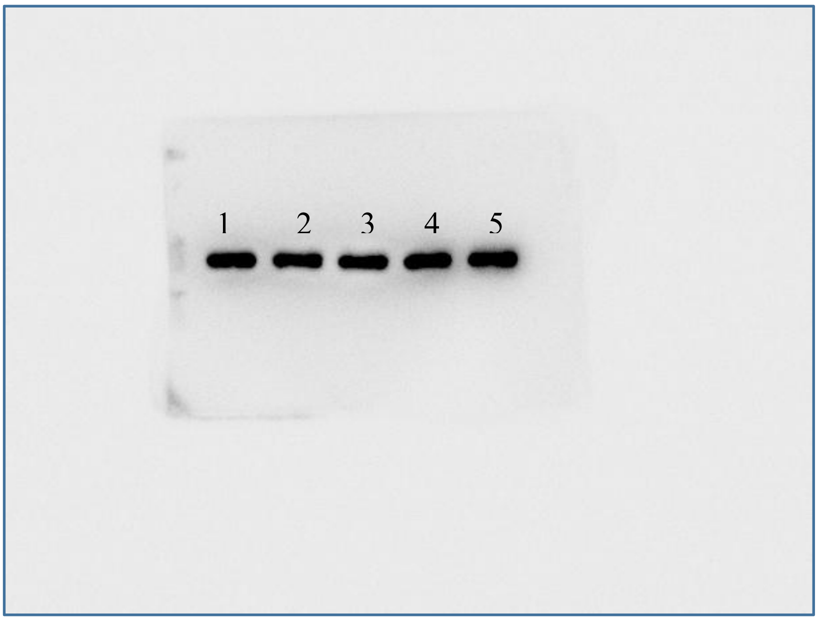

GAPDH-replicate-3

1: Control, 2: Hyporoxia, 3: si-Dvl-1, 4: si-Dvl-1+ Hyporoxia, 5: si-Dvl-1+ Hyporoxia+ MSAB.
